# Biomarkers to predict efficacy of immune checkpoint inhibitors in colorectal cancer patients: a systematic review and meta-analysis

**DOI:** 10.1007/s10238-024-01408-x

**Published:** 2024-07-03

**Authors:** Hang Yu, Qingquan Liu, Keting Wu, Shuang Tang

**Affiliations:** 1 Cancer Institute, Department of Nuclear Medicine, Fudan University Shanghai Cancer Center, 270 Dong-An Road, Shanghai, 200032 People’s Republic of China; 2 School of Clinical Medicine, Shanghai Medical College, Fudan University, Shanghai, 200032 People’s Republic of China; 3 Shanghai Key Laboratory of Radiation Oncology, Shanghai, 200032 People’s Republic of China

**Keywords:** Predictive biomarkers, Immune checkpoint inhibitors, Immunotherapy, Response, Prognosis, Colorectal cancer

## Abstract

Immune checkpoint inhibitors (ICIs) are approved to treat colorectal cancer (CRC) with mismatch-repair gene deficiency, but the response rate remains low. Value of current biomarkers to predict CRC patients’ response to ICIs is unclear due to heterogeneous study designs and small sample sizes. Here, we aim to assess and quantify the magnitude of multiple biomarkers for predicting the efficacy of ICIs in CRC patients. We systematically searched MEDLINE, Embase, the Cochrane Library, and Web of Science databases (to June 2023) for clinical studies examining biomarkers for efficacy of ICIs in CRC patients. Random-effect models were performed for meta-analysis. We pooled odds ratio (OR) and hazard ratio (HR) with 95% confidence interval (CI) for biomarkers predicting response rate and survival. 36 studies with 1867 patients were included in systematic review. We found that a lower pre-treatment blood neutrophil-to-lymphocyte ratio (*n*=4, HR 0.37, 95%CI 0.21–0.67) predicts good prognosis, higher tumor mutation burden (*n*=10, OR 4.83, 95%CI 2.16–10.78) predicts response to ICIs, and liver metastasis (*n*=16, OR 0.32, 95%CI 0.16–0.63) indicates resistance to ICIs, especially when combined with VEGFR inhibitors. But the predictive value of tumor PD-L1 expression (*n*=9, OR 1.01, 95%CI 0.48–2.14) was insignificant in CRC. Blood neutrophil-to-lymphocyte ratio, tumor mutation burden, and liver metastasis, but not tumor PD-L1 expression, function as significant biomarkers to predict efficacy of ICIs in CRC patients. These findings help stratify CRC patients suitable for ICI treatments, improving efficacy of immunotherapy through precise patient management. (PROSPERO, CRD42022346716).

**Supplementary Information:**

The online version contains supplementary material available at 10.1007/s10238-024-01408-x.

## Introduction

Colorectal cancer (CRC) is the second leading cause of cancer-related death, whose survival rate drops sharply to 10% once distant metastases occur [1]. Immune checkpoint inhibitors (ICIs) have made a breakthrough in the fight against cancer. Unfortunately, advanced CRC patients generally respond poorly to ICIs [2]. The ICIs did not get administrative approval to treat CRC patients until DNA mismatch-repair gene deficiency and microsatellite instability-high (dMMR/MSI-H) were identified as predictive biomarkers for efficacy [3]. But still, the response rate to programmed cell death protein 1 (PD1) inhibitors, such as nivolumab and pembrolizumab, is only 30%–45% in dMMR/MSI-H colorectal cancer [4–6]. Moreover, dMMR/MSI-H colorectal cancer only accounts for less than 15% of CRC patients, and this proportion further declines to about 5% in the advanced stage [7, 8]. These two factors result in the fact that CRC patients who can benefit from ICIs are very limited. On the other hand, although patients with microsatellite stable (MSS) tumors in CRC seldom respond to immunotherapy[3], they may achieve tumor regression in case of possessing a DNA polymerase *ε* (POLE) mutation or being treated with combination therapies [9, 10]. Thus, more accurate biomarkers to stratify the patients suitable for ICI treatment are required to improve the outcomes of CRC patients.

Previous meta-analyses focused on the predictors of patient survival or treatment effectiveness with standard therapies [11–13], while systematic studies investigating biomarkers for patients’ prognosis or tumor responses upon ICIs are lacking in CRC patients. Recent clinical trials attempted to bridge objective indicators with clinical benefits from immunotherapies, such as neutrophil-to-lymphocyte ratio (NLR) and tumor mutation burden (TMB) [14–16]. However, the value of these indicators requires systematical evaluation due to heterogeneous populations, small sample sizes, and various study designs. Therefore, we aim to conduct this systematic review and meta-analysis to assess and quantify the magnitude of potential biomarkers that can predict therapeutic efficacy of ICIs in CRC patients.

## Methods

We complied with the Preferred Reporting Items for Systematic reviews and Meta-Analysis (PRISMA) 2020 statement and registered in PROSPERO (CRD42022346716) [17].

### Data sources and searches

We searched databases MEDLINE, Embase, the Cochrane Library, and Web of Science to June 18, 2023. A combination of keywords and free terms related to CRC and ICI were used, along with “survival” or “response.” The full search strategies are presented in Supplementary Table 1.

### Eligibility criteria

The systematic review included studies on CRC patients treated with ICIs written in English or Chinese and published in a peer-reviewed journal. At least one outcome of interest must be reported in the groups with information on biomarkers. We excluded studies without an isolated subgroup of CRC, original data, or those with a sample size of less than ten. Case reports and case series were also excluded. For studies with overlapped patient origin, we chose the one focused on the predictive value of biomarkers. Biomarker changes during treatment were outside our scope.

### Data selection and extraction

After removing duplication in EndNote, version 20.4, two investigators (Liu QQ and Wu KT) independently screened titles and abstracts for relevance. Articles included by either would advance to full-text review. At this stage, three investigators made individual judgments. Disagreements were resolved through discussion.

For each study that reached a consensus for inclusion, one investigator extracted data and another investigator reviewed for accuracy. The data included study characteristics, baseline information of patients, intervention and information related to biomarkers. Biomarkers were collected as categorical variables. Unknown MMR status was treated as proficient mismatch repair (pMMR), which accounts for the majority of metastatic CRC. Outcomes included objective response rate (ORR) and hazard ratio (HR). ORR was calculated as the proportion of patients with complete response or partial response. HR with a 95% confidence interval (CI) based on overall survival (OS) or progression-free survival (PFS) was collected. When data were in doubt, we contacted the authorship for confirmation.

### Quality assessment

Paired investigators evaluated the quality of included studies independently via the Newcastle–Ottawa Scale (NOS) for cohort study [18]. For randomized clinical trials, as only the subgroup that intervened with ICIs was involved, they were evaluated with the same scale. The NOS scores of 0–3, 4–6, and 7–9 indicate low, intermediate, and high quality, respectively. Low-quality studies are more likely to have a high risk of bias.

### Data synthesis and analysis

We use R software with the ‘meta’ package (v.4.20-2) [19] to synthesize data from three or more studies that discussed the same biomarker with a harmonized outcome measure. The odds ratio (OR) was calculated for ORR, and HR was used for survival data. The reciprocals of the HR and its 95%CI were calculated in part of the included studies to make the numerical value have the same clinical meaning. If there were zero events, 0.5 was added to each cell in this study for analysis [20]. Considering the pervasive confounders, we used a random-effect model with the DerSimonian–Laird method to get the pooled estimates for OS, PFS, and ORR [21]. Forest plots were used to display these results.

To investigate a possible heterogenicity, the Cochrane Q test with* I*^*2*^ statistics was used.* I*^*2*^ value of 50% or higher, together with a *p* value less than 0.05, indicate significant heterogenicity. Subgroup analysis was conducted to explain the origin of heterogenicity. To fully discuss the heterogenicity, an article with sufficient information would be divided into two studies based on the MMR status (dMMR or pMMR). Besides, the “leave-one-out” evaluation, a sensitivity analysis, was carried out to verify the stability of the results. Funnel plots and the Egger regression test were performed for publication bias when the number of studies that participated in the pooled estimates was ten or more [22].

## Results

### Search and selection of studies

**Fig. 1:**
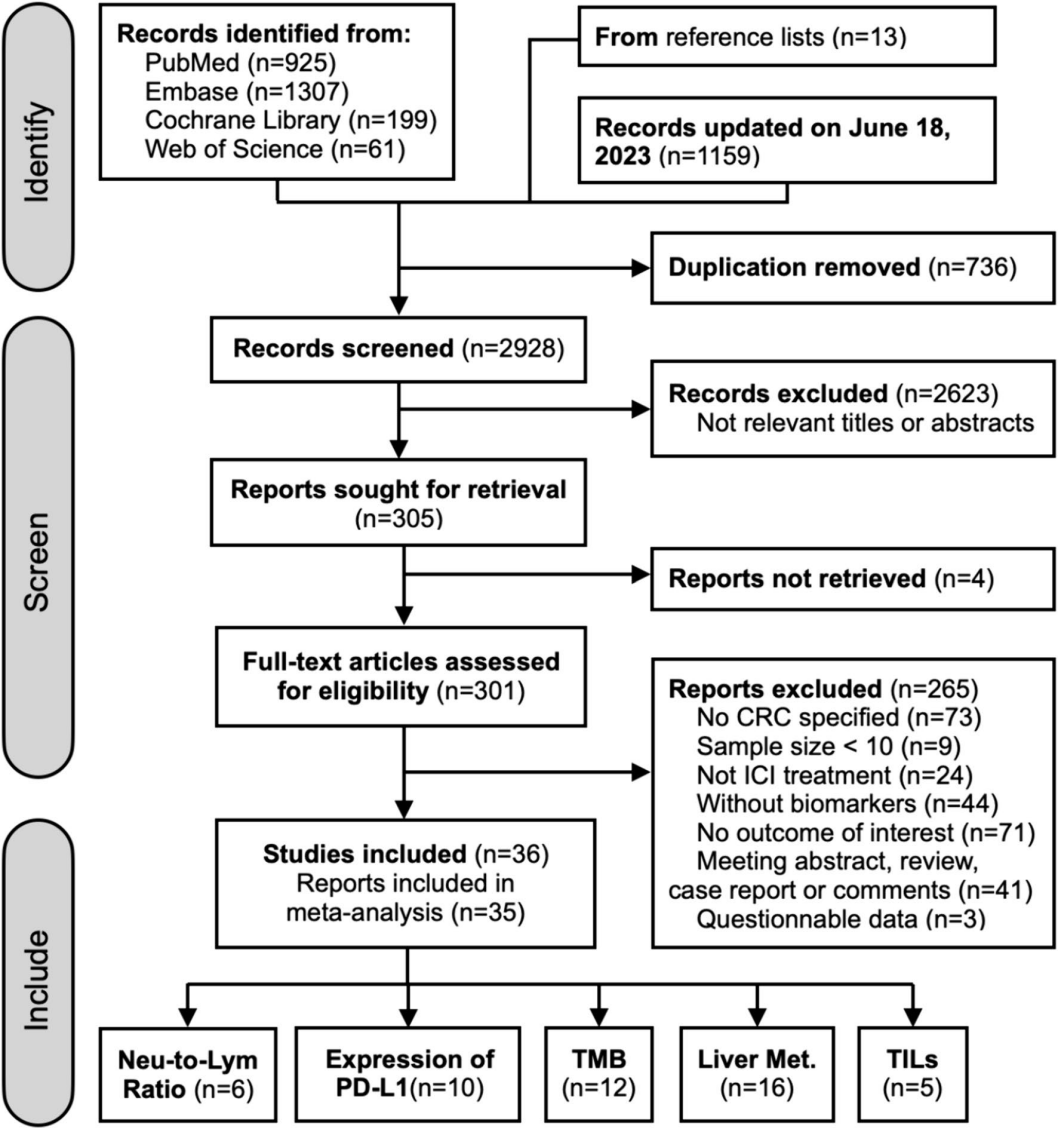
Flow diagram of study selection, compliant with the PRISMA

Among 2928 articles identified from the literature search in MEDLINE, Embase, the Cochrane Library, and Web of Science, 301 full-text articles were assessed for eligibility. A total of 265 studies were excluded because of not specified population (*n*=73) or treatment (*n*=24), without biomarkers (*n*=44) or outcome indicators (*n*=71), small sample size (*n*=9), or article type (*n*=41). Additionally, 3 studies were excluded because their original data could not be accurately extracted and the authors could not be reached [23–25]. At last, 36 studies were eligible for the systematic review, and 35 of them with 1829 patients were included in the meta-analysis (Fig. 1).

### Study characteristics of included studies

The general characteristics are shown in Table 1. All included studies were published from 2015. Sample sizes varied from 10 to 119, with 21 being multi-centered clinical trials. Two studies recruited participants globally [4, 26], and the rest were conducted in East Asia (*n*=15), North America (*n*=14), and Europe (*n*=5).

**Table 1:** General characteristics of the included studies

Study, year	Region	Cases	MMR status	Treatment	Biomarker	Outcome	NOS
Le DT, 2015 [3]	America	32	d:pMMR= 1:2	Anti-PD1	PD-L1 expression TMB, TILs	ORR, OS, PFS	9
Overman MJ, 2017 [4]	Global	74	dMMR	Anti-PD1	PD-L1 expression	ORR	8
O’Neil BH, 2017 [26]	Global	23	pMMR (96%)	Anti-PD1	PD-L1 expression	ORR	5
Schrock AB, 2019 [28]	America	22	dMMR	Anti-PD1	TMB, Liver Met.	ORR, OS, PFS	8
Cheng YK, 2022 [29]	China	41	dMMR	Anti-PD1	NLR	OS	8
Saberzadeh AB, 2023 [30]	America	41	dMMR	Anti-PD1	Liver Met.	ORR, PFS	8
Hyung J, 2022 [31]	Korea	19	dMMR	Anti-PD-(L)1	PD-L1 expression	ORR	7
Jung J, 2023 [32]	Korea	22	NA	Anti-PD-(L)1	TMB	ORR	5
Valero C, 2021 [33]	America	68	NA	Anti-PD1 +/−Anti-PD-L1	NLR, TMB	ORR, OS, PFS	5
Cohen R, 2020 [34]	France	57	dMMR	Anti-PD1 and Anti-CTLA4	(derived) NLR	PFS	9
Loupakis F, 2020 [35]	Italy	80	dMMR	Anti-PD1 +/−Anti-CTLA4	Liver Met., TILs	ORR, OS, PFS	9
Sahin H, 2021 [36]	America	59	dMMR	Anti-PD1 +/−Anti-CTLA4	Liver Met.	ORR	8
Zhou C, 2021 [37]	China	78	dMMR	Anti-PD1 +/ −Anti-CTLA4	TMB	OS	5
Chen EX, 2020 [38]	Canada	119	pMMR	Anti-PD-L1 and Anti-CTLA4	TMB	OS	9
Wang YH, 2022 [39]	China	21	dMMR (76%)	Anti-PD-(L)1 +/−Anti-CTLA4	TMB	ORR	7
Manca P, 2023 [40]	Italy	110	dMMR	Anti-PD-(L)1 +/−Anti-CTLA4	TMB	ORR, OS, PFS	8
Garralada E, 2022 [41]	America	57	pMMR	Anti-PD1 and Anti-LAG3	PD-L1 expression	ORR	8
Fukuoka S, 2020 [42]	Japan	24	pMMR (96%)	Anti-PD1 and VEGFRi	PD-L1 expression TMB, Liver Met.	ORR	7
Li JS, 2020 [43]	China	23	pMMR	Anti-PD1 and VEGFRi	Liver Met.	ORR	7
Yang KL, 2021 [44]	China	84	pMMR	Anti-PD1 and VEGFRi	NLR, Liver Met.	ORR, PFS	7
Li RR, 2022 [45]	China	103	pMMR	Anti-PD1 and VEGFRi	Liver Met.	ORR, OS	7
Xu YJ, 2020 [46]	China	30	pMMR	Anti-PD1 and VEGFRi	Liver Met.	ORR	8
Kim RD, 2022 [47]	America	23	pMMR	Anti-PD1 and VEGFRi	PD-L1 expression Liver Met., TILs	ORR, OS, PFS	9
Fakih M, 2023 [48]	America	29	pMMR	Anti-PD1 and VEGFRi, along with Anti-CTLA4	Liver Met.	ORR	9
Fakih M, 2023 [49]	America	70	pMMR	Anti-PD1 and VEGFRi	Liver Met.	ORR	9
Zhou H, 2021 [50]	China	25	pMMR (84%)	Anti-PD1 and either chemotherapy or VEGFRi	Liver Met.	ORR	5
Mettu NB, 2022 [15]	America	82	pMMR (89%)	Anti-PD-L1 + chemotherapy	Liver Met.	ORR	7
Moretto R, 2023 [51]	Italy	96	pMMR	Anti-PD-L1 + chemotherapy	PD-L1 expression TILs	ORR, PFS	8
Bando H, 2022 [27]	Japan	38	pMMR	(neoadjuvant) Anti-PD1 + chemoradiotherapy	TILs	ORR	8
Parikh AR, 2021 [10]	America	10	pMMR	Anti-PD1 and Anti-CTLA4 with radiation therapy	TMB	ORR	6
Ciardiello D, 2022 [57]	Italy	77	pMMR (92%)	Anti-PD-L1 + cetuximab	NLR	ORR, OS, PFS	7
Kawazoe A, 2020 [52]	Japan	50	d:pMMR=1:4	Anti-PD1 and STATi	PD-L1 expression	ORR	8
Chida K, 2021 [53]	Japan	17	dMMR	Anti-PD1 +/– STATi	TMB	ORR	8
Kim DW, 2021 [54]	America	38	pMMR	Anti-PD1 and BTKi	NLR	ORR, OS, PFS	8
Kawazoe A, 2021 [55]	Japan	29	pMMR (86%)	Anti-PD1 and HSP90i	PD-L1 expression TMB, Liver Met.	ORR	7
Wang CK, 2021 [56]	America	96	pMMR	Anti-PD-(L)1 +/– other investigational agents	Liver Met.	ORR, PFS	8

*dMMR/pMMR* deficient/proficient mismatch repair protein; *TMB* tumor mutation burden; *TILs* tumor-infiltrating lymphocytes; *NLR* neutrophils-to-lymphocytes ratio; *ORR* objective response rate; *OS* overall survival; *PFS* progression-free survival; *NA* not available; *NOS* Newcastle–Ottawa Scale; *PD1* programmed cell death protein 1; *PD-L1* programmed death ligand 1; *CTLA-*4 cytotoxic T-lymphocyte-associated protein 4; *LAG*-3 lymphocyte-activation gene 3; *VEGFRi* vascular endothelial growth factor

Most included studies focused on metastatic CRC patients, except one focused on rectal cancer in locally advanced stage [27]. Tumors with pMMR/MSS characteristic comprised the majority in most studies (*n*=20), whereas dMMR/MSI-H tumors were required in 12 studies. For treatment strategies, there were 8 studies focused on ICI monotherapy [3, 4, 26, 28–32], all of which were anti-PD1 therapy, and 9 studies permitted or applied dual ICI therapy [33–41]. Moreover, the majority of the studies applied combined therapies with ICIs, involving vascular endothelial growth factor receptor inhibitors (VEGFRi, *n*=9) [42–50], chemoradiotherapy (*n*=5) [10, 15, 27, 50, 51], other investigational agents (*n*=5) [52–56] and Cetuximab, an epidermal growth factor receptor blockade (*n*=1) [57]. Detailed information can be achieved from Supplementary Table 2.

Most of the included studies were at low risk of bias (30 out of 36, Table 1), while the rest owned a moderate risk, mainly attributed to their retrospective study design and lack of control for confounding factors. The NOS scores are presented in Supplementary Table 3 in detail.

### Low pretreatment blood neutrophil-to-lymphocyte ratio (NLR) predicts good prognosis for CRC patients upon ICIs

NLR is the absolute neutrophil count divided by the absolute lymphocyte count obtained from the blood count [54, 57]. Pre-treatment NLR was assessed in 6 studies [29, 33, 34, 44, 54, 57], with cutoffs ranging from 1.5 to 5.

**Fig. 2:**
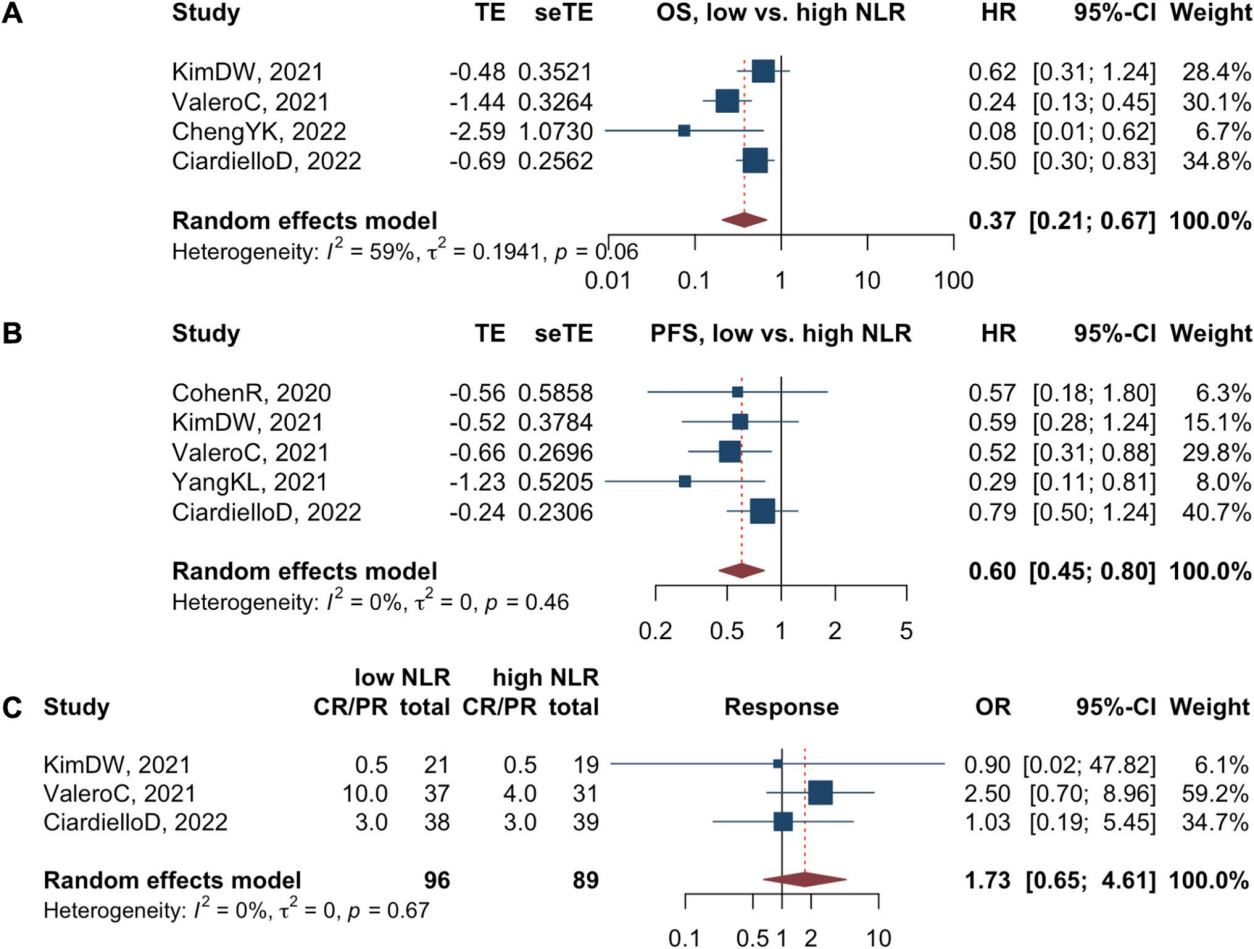
Forest plots for pretreatment blood neutrophil-to-lymphocyte ratio (NLR) on overall survival (**A**), progression-free survival (**B**), and objective response rate (**C**) in CRC patients treated with ICIs

For CRC patients treated with ICIs, those with a low pretreatment NLR show less risk of death than those with a high pretreatment NLR (n=4 studies, HR 0.37, 95%CI 0.21–0.67,* I*^2^=59%, *p*=0.06)(Fig. 2A) [29, 33, 54, 57], with robustness verified (Supplementary Fig. 1A). The subgroup analysis (Table 2) showed that the moderate heterogenicity could be attributed to different treatment strategies. Besides, using 5 as the cutoff value may be more efficient than a value less than 5.

**Table 2:** Results of the subgroup analysis

Item	Subgroup	Num.	HR/OR (95%CI)	I2 (*p* value)
*Neutrophil-to-lymphocyte ratio (low vs. high)–Overall survival*				
			0.37 (0.21–0.67)	59% (0.06)
Treatment	Immune checkpoint inhibitors^a^	2	0.21 (0.11–0.42)	5% (0.31)
	Combined treatment^b^	2	0.54 (0.36–0.81)	0% (0.62)
Cutoffs	= 5	2	0.38 (0.15–0.98)	75% (0.05)
	< 5	2	0.26 (0.04–1.51)	66% (0.09)
*Expression of PD-L1 (high vs. low)–Objective response rate*				
			1.01 (0.48–2.14)	19% (0.26)
Definition	Tumor positive score	3	0.85 (0.27–2.67)	37% (0.19)
	Combined positive score	5	0.98 (0.34–2.81)	6% (0.38)
MMR status	Deficient (dMMR)	4	0.56 (0.23–1.39)	0% (0.72)
	Proficient (pMMR)	7	1.65 (0.58–4.68)	22% (0.26)
*Tumor Mutation Burden (high vs. low)–Objective response rate*				
			4.83 (2.16–10.78)	24% (0.23)
MMR status	Deficient (dMMR)	3	7.63 (1.37–42.65)	35% (0.21)
	Proficient (pMMR)	3	2.59 (0.62–10.72)	0% (0.84)
	Both deficient and proficient	4	5.25 (0.96–28.72)	60% (0.06)
Cutoffs	> 20 mutations/Mb	5	5.19 (1.80–14.97)	20% (0.29)
	<= 20 mutations/Mb	5	4.22 (1.02–17.36)	41% (0.15)
*Presence of liver metastasis (with vs. without)–Progression-free survival*				
			2.26 (1.34–3.83)	65% (< 0.01)
Region/Treatment	Western/Immune checkpoint inhibitors	4	2.27 (0.97–5.33)	74% (< 0.01)
	Eastern/Combined with VEGFRi	2	2.02 (1.30–3.14)	0% (0.33)
*Liver metastasis (with vs. without)–Objective response rate*				
			0.32 (0.16–0.63)	44% (0.03)
Sample size	> 50	8	0.37 (0.10–1.35)	52% (0.04)
	<= 50	8	0.30 (0.13–0.63)	44% (0.08)
Region	Western	8	0.26 (0.11–0.61)	48% (0.06)
	Eastern	8	0.45 (0.13–1.53)	48% (0.06)
MMR status	Deficient (dMMR)	3	0.41 (0.11–1.50)	68% (0.04)
	Proficient (pMMR)	11	0.22 (0.11–0.46)	0% (0.64)
	Both deficient and proficient	2	1.65 (0.02–130.69)	89% (< 0.01)
Treatment	Immune checkpoint inhibitors	4	0.44 (0.16–1.18)	52% (0.10)
	Combined with VEGFRi	8	0.23 (0.10–0.54)	0% (0.63)
	Other combined treatment	4	0.37 (0.03–4.64)	77% (< 0.01)

*HR* hazard ratio; *OR* odds ratio; *CI* confidence interval; *PD-L1* programmed death ligand 1; *MMR* mismatch-repair protein; *VEGFRi* vascular endothelial growth factor receptor inhibitor

^a^ immune checkpoint inhibitor alone is mainly used among dMMR/MSI-H colorectal cancer

^b^ combined treatment is mainly used among pMMR/MSS colorectal cancer

Furthermore, low pretreatment NLR predicted a slow disease progression of CRC during the treatment of ICIs (*n*=5 studies, HR 0.60, 95%CI 0.45– 0.80, *I*^2^=0%, *p*=0.46) (Fig. 2B) [33, 34, 44, 54, 57]. According to sensitivity analysis, NLR derived from the difference between leukocytes and neutrophils could be used as a substitute (HR 0.59, 95%CI 0.42–0.83)(Supplementary Figure 1B) [34].

However, the pooled OR for response to treatment was 1.73 (*n*=3 studies, 95% CI 0.65–4.61, low versus high NLR, *I*^2^=0%, *p*=0.67)(Fig. 2C) [33, 54, 57], suggesting that a pre-treatment low NLR showed a trend but was insufficient to predict shrinkage of tumor upon ICI treatment.

### Tumor PD-L1 expression in predicting response of CRC patients upon anti-PD-1/PD-L1 therapy

**Fig. 3:**
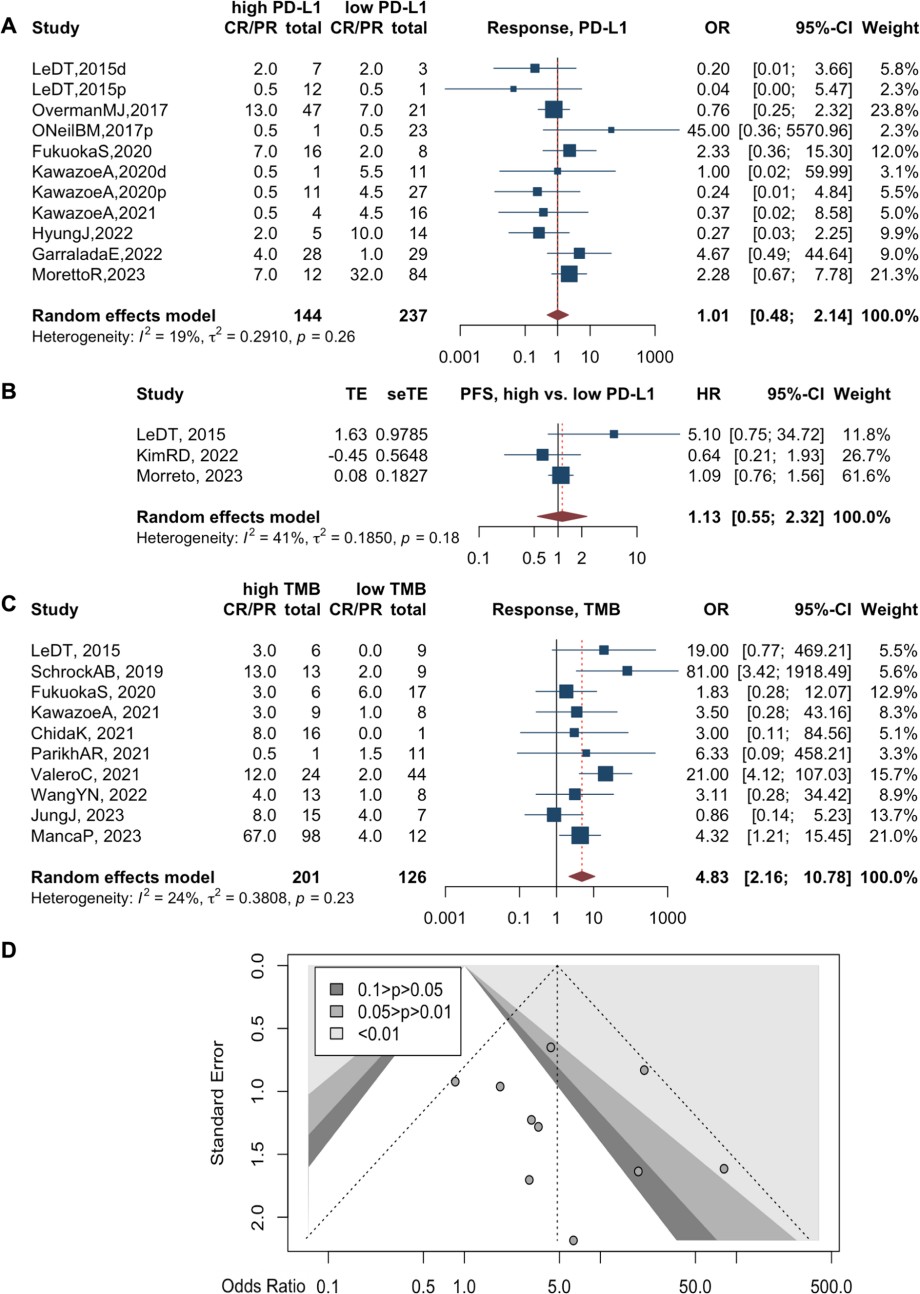
Forest plots for PD-L1 expression on objective response rate (**A**) and progression-free survival (**B**). Forest plots for tumor mutation burden (TMB) on objective response rate (**C**). Funnel plot for publication bias regarding TMB in CRC patients treated with ICIs (**D**)

We included 9 studies using high or positive expression of tumor PD-L1 as a biomarker for ICIs [3, 4, 26, 31, 41, 42, 51, 52, 55]. However, PD-L1 expression was insufficient to predict tumor response to ICI treatment (OR 1.01, 95%CI 0.48–2.14, high versus low expression, *I*^2^=19%, *p*=0.26)(Fig. 3A, Supplementary Figure 1C). Of note, two criteria were reported to evaluate the level of PD-L1 expression in studies. The combined positive score (CPS) was used in 5 studies (OR 0.98, 95%CI 0.34–2.81) [31, 41, 42, 52, 55], calculated by the count of PD-L1 positive tumor cells and immune cells divided by the total number of tumor cells multiplied by 100. Tumor tissue with CPS>1 was considered high or positive in PD-L1 expression. Additionally, 3 articles (OR 0.85, 95%CI 0.27–2.67) used a 5% cutoff value to count the PD-L1 on tumor cells, separating from immune cells [3, 4, 51]. Neither of these evaluation criteria could rescue the inadequate predictive value of tumor PD-L1 expression for ICI treatment in CRC patients (Table 2).

In terms of disease progression, a high expression of PD-L1 in tumor tissue seems to be a mild but not significant risk factor for CRC patients under the situation of anti-PD therapy (*n*=3 studies, HR 1.13, 95%CI 0.55–2.32, *I*^2^=41%, *p*=0.18) (Fig. 2B, Supplementary Figure 1D) [3, 47, 51].

### Tumor mutation burden (TMB) predicts efficacy of immunotherapy in CRC patients

We enrolled 12 studies relevant to TMB. TMB is generally defined as the number of somatic non-synonymous mutations in the tumor tissue derived from the NGS as recommended, sometimes with synonymous mutations [28, 39]. A few cases also used the whole exome sequencing technique to detect TMB [10, 53].

CRC patients with a TMB-high tumor are more likely to respond to ICI treatment compared with a TMB-low tumor (*n*=10 studies, OR 4.83, 95%CI 2.16–10.78, *I*^2^=24%, *p*=0.23) (Fig. 3C, Supplementary Figure 1E) [3, 10, 28, 32, 33, 39, 40, 42, 53, 55]. Of note, the cutoffs varied across studies, ranging from 9.6 to 41 mutations/Mb. We considered a value above 20 mutations/Mb as high to maintain a consistent number between subgroups. The corresponding pooled OR was 5.19 (95%CI 1.80–14.97) and 4.22 (95%CI 1.02–17.36) for high and low cutoffs (Table 2), suggesting that cutoffs do not affect the predictive value of TMB in CRC patients. Egger’s test for funnel plot asymmetry indicated no significant publication bias (*p*=0.58) (Fig. 3D). As a previous meta-analysis had fully discussed the role of TMB in predicting survival in CRC patients treated with ICIs [58], we did not repeat the test here.

### The presence of liver metastasis (LM) predicts resistance to ICIs for CRC patients

Data related to colorectal liver metastasis upon ICI treatment in CRC patients were extracted from 16 articles. Notably, when CRC co-exists with metastatic lesions in the liver, patients owned a high risk of death (*n*=4 studies, HR 1.64, 95%CI 1.11–2.42, with versus without LM, *I*^2^=0%, *p*=0.48) (Supplementary Figure 2A, 2B) [28, 35, 45, 47]. Moreover, CRC patients with LM were more likely to suffer from disease progression even under the treatment of ICIs (*n*=6 studies, HR 2.26, 95%CI 1.34–3.83, *I*^2^=65%, *p*=0.01) compared with patients without LM (Supplementary Figure 2C) [28, 30, 35, 44, 47, 56], associated with a significant moderate heterogenicity. The sensitivity analysis proved the robustness of LM as a risk factor (Supplementary Figure 2D). Regrettably, our subgroup analysis could not fully explain the origin of heterogenicity (Table 2).

**Fig. 4:**
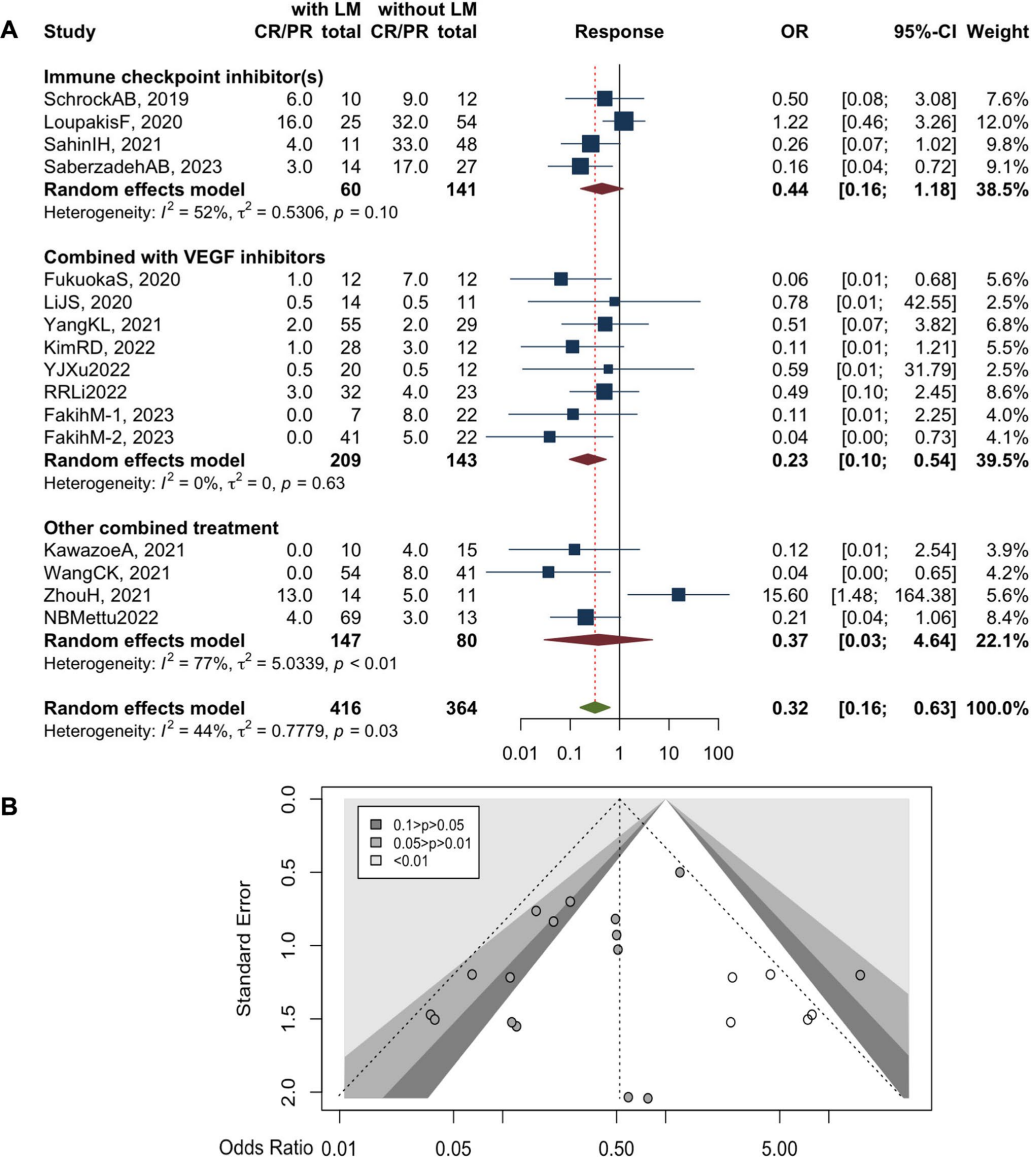
Forest plots for liver metastasis on objective response rate (**A**), stratified by the treatment strategies. Funnel plot for publication bias regarding liver metastasis in CRC patients treated with ICIs (**B**)

Next, we analyzed the value of LM for predicting tumor response to ICI treatment among CRC patients. The pooled OR was 0.32 (*n*=16 studies, 95%CI 0.16–0.63, with versus without LM, *I*^2^=44%, *p*=0.03) with a mild heterogenicity (Fig. 4A). Sensitivity analysis proved its robustness (Supplementary Figure 2E). We identified a subgroup treated with an anti-PD1 agent and VEGFR inhibitor in combination, in which CRC patients with LM are much more likely to resist the therapy (*n*=8 studies, OR 0.23, 95%CI, 0.10–0.54, *I*^2^=0%, *p*=0.63) (Fig. 4A) [42–49]. The funnel plot was displayed (Fig. 4B) and Egger’s test indicated no significant publication bias with a *p* value of 0.20. Taken together, the presence of LM in CRC patients predicts a poor prognosis and resistance to ICI treatment, especially when in combination with anti-VEGFR agents.

### Tumor-infiltrating lymphocytes (TILs) in association with prognosis of CRC and response to ICIs

We reviewed 5 studies on the predictive value of TILs, which were not amenable to the meta-analysis due to different identities. Two studies counted lymphocytes infiltrating in tumor epithelium, leaving out those in the stroma [35, 51]. When the cutoff value is 2.0, one study showed a predictive value of TILs in response and survival [35], while the other reported no difference between TILs-high and TILs-low groups in disease progression [51]. Three studies focused on the density of CD8^+^ T lymphocytes and T_reg_ lymphocytes [3, 27, 47]. One study found a higher pathological complete response rate with a greater CD8/T_reg_ ratio cutoff of 2.5 (*p*=0.003) [27].

## Discussion

How to predict tumor response to immunotherapy and select patients who could benefit from ICIs remain a major clinical challenge in colorectal cancer. Here, we evaluated multiple biomarkers that could predict the efficacy of ICI treatment in CRC patients. Following the PRISMA statement, we provided strong and objective evidence that a low pre-treatment blood NLR was a protective predictor for CRC patients. Moreover, high TMB in tumor tissue predicts response while liver metastasis predicts resistance to ICIs in CRC patients. The predictive value of PD-L1 expression was insignificant. This study covered several biological markers of interest in the clinic and discussed them under various situations, such as different MMR statuses and treatment strategies.

Peripheral immune cells are regarded as one of the hallmarks of response to ICIs from the perspective of systemic immunity [59], with advantages for its ease of assessment. We validated its prognostic value in the case of ICI treatment in terms of OS and PFS, in line with the results in melanoma and lung cancer treated with ICIs [60, 61]. The survival benefit as measured by overall survival was more significant in patients treated with ICI single therapy (mainly among dMMR/MSI-H colorectal cancer) than in patients who received ICI combined with another inhibitor (mainly among pMMR/MSS colorectal cancer) (HR 0.21 vs. 0.54). Considering the different cutoffs, a higher NLR was associated with poorer overall survival across all decile cutoffs [33]. Compared to lymphocytes primarily responsible for antitumor immunity, the role of neutrophils in tumor immunity is heterogeneous. Neutrophils in circulation have an influence on the number of tumor-associated neutrophils in tumor microenvironment (TME), which contributes to tumor progression in multiple ways, such as amplifying DNA damage through the release of reactive oxygen species and inducing T cell exhaustion through express PD-L1 expression [62].

LM is a strong risk factor for CRC patients undergoing ICI treatment. Recently, LM has been reported to inhibit immunotherapy's efficacy via macrophage-mediated T-cell elimination [63]. Also, the immunosuppressive TME within the metastatic sites further declines the response to immunotherapy [64, 65]. Notably, when combined with VEGFR inhibitors such as regorafenib, patients without LM were more sensitive to ICI treatment with an ORR four times higher than those with LM. Interestingly, two included studies reported that patients with a history of surgery or intervention for LM benefit from combination treatment in survival and response [43, 56]. They suggested that liver resection or radiofrequency ablation before ICI treatment could promote the likelihood of patients’ response to dual-agent therapy with ICIs and VEGFR inhibitors. Larger and well-designed clinical trials are required to investigate the impact of LM treatment on CRC patients’ response to ICI treatment.

Furthermore, our studies investigated TMB and PD-L1 expression as biomarkers closely related to TME, which is a crucial mediator of cancer progression and closely involved in tumor response to therapy [66]. For example, the pH of TME suggests us to alkalize the acidic TME, which may improve the function of immune cells and sensitivity to anticancer drugs [67]. As a tumor-intrinsic feature that reflects cancer mutation quantity, TMB reflects tumor foreignness and is related to tumor neo-antigens within TME, which are presented by major histocompatibility complex proteins to T cells [68]. Therefore, TMB is expected to drive anti-tumor immunity and predict tumor responses to immunotherapy. Indeed, our meta-analysis showed that TMB consistently predicts the response to ICIs in dMMR/MSI-H colorectal cancer, with some variation in magnitude compared to a previous meta-analysis [58]. Of note, TMB was insignificant in predicting tumor responses to immunotherapy among patients with pMMR/MSS colorectal cancer. Regarding the cutoffs, when the TMB median is within a normal range, an institution-specific cutoff is acceptable [69]. However, the TCGA lower bound of CRC hypermutated phenotype is 12 mutations/Mb [70], and another meta-analysis suggests a TMB of 12.3 mutations/Mb as the best cutoff in CRC for immunotherapy [71]. Since TMB was taken as a dichotomous biomarker, we observe no difference in clinical significance between groups with high and low cutoffs [72].

Besides, the expression of immune inhibitory PD-L1 protein on antigen-presenting cells and tumor cells within TME can attract immune cells with its ligand PD1, which is broadly expressed on effector memory T cells from the peripheral blood and lymphoid tissue [73]. Although PD-L1 expression can predict the response to ICIs in non-small cell lung cancer [74], such an effect was not achieved in CRC. This result could be partly explained by the overall low expression of PD-L1 in the tumor tissue of CRC compared to other tumors and the existence of programmed death ligand 2 [72].

Our study has several strengths. Firstly, it is a meta-analysis stock on the PRISMA standard. Secondly, this study objectively analyzed a range of pre-treatment biomarkers to predict efficacy under ICI treatment in CRC patients. Thirdly, differences in definitions and cutoffs of biomarkers were considered and adjusted by subgroup analysis.

Still, our study has several limitations. Firstly, the patient population was complex, and we were unable to adjust for previous treatment and gene background due to a lack of detailed information. Secondly, half studies had a small sample size and retrospective designs. The situation of limited cases is aggravated by dealing with biomarkers that sample from tumor tissue.

In conclusion, beyond MMR status, lower NLR is a biomarker that predicts better survival, TMB is a predictive biomarker for tumor response, while liver metastasis is a biomarker for resistance in CRC patients upon ICI treatment. These findings help stratify CRC patients suitable for ICI treatments, improving the efficacy of ICIs by precise CRC patient management.

## Supplementary Information

Below is the link to the electronic supplementary material.

Supplementary file1 (DOCX 1296 KB)

## Data Availability

Data, analytic methods, and study materials will be available.
