# Supplementary material for: Biomarkers to predict efficacy of immune checkpoint inhibitors in colorectal cancer patients: a systematic review and meta-analysis
Source: Clin Exp Med. 2024 Jul 3;24(1):143. doi: 10.1007/s10238-024-01408-x (PMC11222262; doi:10.1007/s10238-024-01408-x)
Supplement: Supplementary file 1 — Supplementary file1 (DOCX 1296 KB) [file 10238_2024_1408_MOESM1_ESM.docx]

**Supplementary materials**

**CONTENT**

Supplementary Table 1 Search Strategies

Supplementary Table 2 Information of included studies

Supplementary Table 3 Quality Assessment with Newcastle-Ottawa Scale

Acronym Table for this review

Supplementary Figure 1 Sensitivity Analysis

Supplementary Figure 2 Results of liver metastasis

**Supplementary Table 1 Search Strategies**

PubMED

| 1 | colorectal neoplasms[MeSH Terms] |
| --- | --- |
| 2 | (((((((((((((((colorectal neoplasm[Title/Abstract]) OR (colorectal cancer[Title/Abstract])) OR (colorectal carcinoma[Title/Abstract])) OR (colorectal tumor[Title/Abstract])) OR (colon neoplasm[Title/Abstract])) OR (colon cancer[Title/Abstract])) OR (colon carcinoma[Title/Abstract])) OR (colon tumor[Title/Abstract])) OR (colonic neoplasm[Title/Abstract])) OR (colonic cancer[Title/Abstract])) OR (colonic carcinoma[Title/Abstract])) OR (colonic tumor[Title/Abstract])) OR (rectal neoplasm[Title/Abstract])) OR (rectal cancer[Title/Abstract])) OR (rectal carcinoma[Title/Abstract])) OR (rectal tumor[Title/Abstract]) |
| 3 | #1 OR #2 |
| 4 | immune checkpoint inhibitors[MeSH Terms] |
| 5 | (((((((((((immune checkpoint inhibitor) OR (immune checkpoint inhibition)) OR (immune checkpoint blocker)) OR (immune checkpoint blockade)) OR (PD1 inhibitor)) OR (PD1 blockade)) OR (programmed cell death protein 1 inhibitor)) OR (PD-L1 inhibitor)) OR (PD-L1 blockade)) OR (programmed death ligand 1 inhibitor)) OR (CTLA-4 inhibitor)) OR (CTLA-4 blockade)) OR (cytotoxic T-lymphocyte-associated protein 4 inhibitor) |
| 6 | ((((((((((((((nivolumab) OR (Opdivo)) OR (pembrolizumab)) OR (Keytruda)) OR (cemiplimab)) OR (Libtayo)) OR (tislelizumab)) OR (atezolizumab)) OR (Tecentriq)) OR (avelumab)) OR (Bavencio)) OR (durvalumab)) OR (Imfinzi)) OR (ipilimumab)) OR (Yervoy) |
| 7 | #4 OR #5 OR #6 |
| 8 | (survival) OR (response) |
| 9 | (#3 AND #7 AND #8) NOT (review[Filter]) |

EMBASE

| 1 | ‘colorectal carcinoma’/exp OR ‘colorectal cancer’/exp OR ‘colorectal tumor’/exp |
| --- | --- |
| 2 | ‘colorectal carcinoma’:ti,ab,kw OR ‘colorectal cancer’:ti,ab,kw OR ‘colorectal tumor’:ti,ab,kw OR ‘colorectal neoplasm’:ti,ab,kw OR ‘colon carcinoma’:ti,ab,kw OR ‘colon cancer’:ti,ab,kw OR ‘colon tumor’:ti,ab,kw OR ‘colon neoplasm’:ti,ab,kw OR ‘rectal carcinoma’:ti,ab,kw OR ‘rectal cancer’:ti,ab,kw OR ‘rectal tumor’:ti,ab,kw OR ‘rectal neoplasm’:ti,ab,kw OR ‘colonic carcinoma’:ti,ab,kw OR ‘colonic cancer’:ti,ab,kw OR ‘colonic tumor’:ti,ab,kw OR ‘colonic neoplasm’:ti,ab,kw |
| 3 | #1 OR #2 |
| 4 | 'immune checkpoint inhibitor'/exp |
| 5 | 'immune checkpoint inhibitor':ti,ab,kw OR 'immune checkpoint inhibition':ti,ab,kw OR 'immune checkpoint blocker':ti,ab,kw OR 'immune checkpoint blockade':ti,ab,kw OR 'pd1 inhibitor':ti,ab,kw OR 'pd1 blockade':ti,ab,kw OR 'programmed cell death protein 1 inhibitor':ti,ab,kw OR 'pd-l1 inhibitor':ti,ab,kw OR 'pd-l1 blockade':ti,ab,kw OR 'programmed death ligand 1 inhibitor':ti,ab,kw OR 'ctla-4 inhibitor':ti,ab,kw OR 'ctla-4 blockade':ti,ab,kw OR 'cytotoxic t-lymphocyte-associated protein 4 inhibitor':ti,ab,kw |
| 6 | 'nivolumab' OR 'opdivo' OR 'pembrolizumab' OR 'keytruda' OR 'tislelizumab' OR 'atezolizumab' OR 'tecentriq' OR 'avelumab' OR 'bavencio':ti,ab,kw OR 'durvalumab' OR 'imfinzi' OR 'cemiplimab' OR 'libtayo' OR 'ipilimumab' OR 'yervoy' |
| 7 | #4 OR #5 OR #6 |
| 8 | #3 AND #7 AND (‘survival’ OR ‘response’) |
| 9 | #9 AND ‘article’/it |

Web of Science

| 1 | TS=(colorectal neoplasm OR colorectal carcinoma OR colorectal cancer OR colorectal tumo$r OR colon neoplasm OR colon carcinoma OR colon cancer OR colon tumo$r OR rectal neoplasm OR rectal carcinoma OR rectal cancer OR rectal tumo$r OR colonic neoplasm OR colonic carcinoma OR colonic cancer OR colonic tumo$r) |
| --- | --- |
| 2 | TS=(immune checkpoint inhibitor OR immune checkpoint inhibition OR immune checkpoint blocker OR immune checkpoint blockade OR PD 1 inhibitor OR PD 1 blockade OR programmed cell death protein 1 inhibitor OR PD-L1 inhibitor OR PD-L1 blockade OR programmed death ligand 1 inhibitor OR CTLA-4 inhibitor OR CTLA-4 blockade OR cytotoxic T-lymphocyte-associated protein 4 inhibitor) |
| 3 | TS=(nivolumab OR pembrolizumab OR atezolizumab OR avelumab OR durvalumab OR tislelizumab OR cemiplimab OR Opdivo OR Keytruda OR Bavencio OR Imfinzi OR Libtayo OR ipilimumab OR Yervoy) |
| 4 | TS=(survival OR response) |
| 5 | #2 OR #3 |
| 6 | #1 AND #5 AND #4 |
| Limits | Language: English or Chinese, NOT Database: MEDLINE, NOT Document types: patent or meeting or review or editorial or books or revise or online |

The Cochrane Library

| 1 | MeSH descriptor: [Colorectal Neoplasms] explode all trees |
| --- | --- |
| 2 | ((colorectal neoplasm) OR (colorectal carcinoma) OR (colorectal cancer) OR (colorectal tumor)):ti,ab,kw |
| 3 | ((colon neoplasm) OR (colon carcinoma) OR (colon cancer) OR (colon tumor)):ti,ab,kw |
| 4 | ((rectal neoplasm) OR (rectal carcinoma) OR (rectal cancer) OR (rectal tumor)):ti,ab,kw |
| 5 | ((colonic neoplasm) OR (colonic carcinoma) OR (colonic cancer) OR (colonic tumor)):ti,ab,kw |
| 6 | #1 OR #2 OR #3 OR #4 OR #5 |
| 7 | MeSH descriptor: [Immune Checkpoint Inhibitors] explode all trees |
| 8 | ((Immune checkpoint inhibitor) OR (immune checkpoint blocker) OR (immune checkpoint inhibition) OR (immune checkpoint blockade)):ti,ab,kw |
| 9 | ((PD1 inhibitor) OR (PD1 blockade) OR (programmed cell death protein 1 inhibitor)):ti,ab,kw |
| 10 | ((PD L1 inhibitor) OR (PD L1 blockade) OR (programmed death protein 1 inhibitor)):ti,ab,kw |
| 11 | ((CTLA-4 inhibitor) OR (CTLA-4 blockade) OR (cytotoxic T-lymphocyte-associated protein 4 inhibitor):ti,ab,kw |
| 12 | (nivolumab OR pembrolizumab OR atezolizumab OR avelumab OR durvalumab OR tislelizumab OR cemiplimab OR ipilimumab):ti,ab,kw |
| 13 | (Opdivo OR Keytruda OR Tecentriq OR Bavencio OR Imfinzi OR Libtayo OR Yervoy):ti,ab,kw |
| 14 | #7 OR #8 OR #9 OR #10 OR #11 OR #12 OR #13 |
| 15 | (survival OR response):ti,ab,kw |
| 16 | #6 AND #14 AND #16 |

**Supplementary Table 2 Information of included studies**

| **Study** | **Characteristics of patients** | **Features of tumor** | **Treatment** | **Biomarker (Definition, test method, cutoffs)** | **Notes** |
| --- | --- | --- | --- | --- | --- |
| Publication year: 2015 | | | | | |
| Le, D.T.  America  Multi-centered  Prospective  Phase 2  NCT01876511 | Adults with histologically confirmed evidence of previously treated, progressive carcinoma.  Age (range): 24-79  Sex: Female: Male = 13:19  Race: White (25), Black (4)  Previous treatment: two lines (7), three (8), more (17) | dMMR:pMMR =11:21, assessed with the MSI Analysis System. Tumors were designated as MSI if two or more mononucleotide loci varied in length compared to the germline DNA.  Location: Colon (27), Rectum (5)  Metastasis: 17 with liver met.  **Lynch** Y:N:U = 9:23:0  **BRAF mut** Y:N:U = 1:19:12  **KRAS mut** Y:N:U = 13:19:0 | Pembrolizumab (anti-PD1), 10mg/kg every 14 days. | **PD-L1 expression** high: fraction of malignant cells ≥ 5%  Fraction of malignant cells exhibiting a membranous pattern of PD-L1 expression at the invasive front, detected by IHC and quantified by three pathologists. | Primary endpoints: ORR and 20-week PFS  Criteria: RECIST 1.1 and immune-related response criteria  Radiographic assessments were performed at 12 weeks and every 8 weeks thereafter.  Follow-up: 12 months |
|  |  |  |  | **Tumor mutation burden** (mutations)  Mutations that meet four criteria (see the original article), detected by WES and then identified using VariantDx custom software  ** Divide by 35 to transfer the unit into mutations/Mb* |  |
|  |  |  |  | **Tumor-infiltrating lymphocytes** (cells/mm^2^)  The CD8-stained cells in the regions corresponding to tumor and invasive front, detected with IHC and calculated the density using a custom algorithm implemented in PIP |  |
| Publication year: 2017 | | | | | |
| Overman, M.J.  Global  Multi-centered  Prospective  Phase 2  NCT02054806 CheckMate-142 | Adults with histologically confirmed recurrent or metastatic CRC locally assessed as dMMR/MSI-H.  Age (median): 52.5  Sex: Female: Male = 44:30  Race: White (65), Black (7)  Previous treatment: no (1), one line (11), two (22), more (40) | dMMR/MSI-H, assessed with PCR (modified Bethesda panel including TGFβR type 2) centrally. Tumor samples with instability in two or more markers were identified as MSI-H.  **Lynch** Y:N:U = 27:28:19  **BRAF mut** Y:N:U = 12:55:7  **KRAS mut** Y:N:U = 26:41:7 | Nivolumab (anti-PD1) was given at a dose of 3mg/kg every 14 days | **PD-L1 expression** high: positive staining ≥ 1%  Positive staining was defined as complete circumferential or partial linear plasma membrane staining, detected by PD-L1 IHC 28-8 pharmDx assay | Primary endpoints: ORR  Criteria: RECIST 1.1  Radiographic assessments were performed at baseline, every 6 weeks for 24 weeks, and every 12 weeks thereafter.  Follow-up: 12 months |
| O’Neil, B.H.  Global  Multi-centered  Prospective  Phase 1b  NCT02060188, KEYNOTE-028 | Patients with advanced, treatment-resistant CRC with PD-L1 expression positive.  Age: median 57, range 40-78  Sex: Female: Male = 10:13  Race: White (11), Asian (6)  Previous treatment: no (1), two (7), three (7), more (8) | dMMR:pMMR = 1:22, which is not specified in the protocol, and retrospectively determined by the investigator.  Location: Colon (16), Rectum (5), Others (2) | Pembrolizumab (anti-PD1), 10mg/kg every 14 days. | **PD-L1 expression** high: positive  Positivity was defined as membrane staining in >= 1% of scorable cells or the presence of a distinctive interface pattern in neoplastic cells and contiguous mononuclear inflammatory cells, detected by IHC using the 22C3 antibody on either archival or pre-treatment study biopsy samples | Primary endpoints: safety, ORR  Criteria: RECIST 1.1  Radiographic assessments were performed every 8 weeks for 6 months, and every 12 weeks thereafter.  Follow-up: 12 months |
| Publication year: 2019 | | | | | |
| Schrock, A.B.  America  Multi-centered  Retrospective | Metastatic CRC with MSI-H status  Age: median 32, range 29-91  Sex: Female: Male = 12:10 | dMMR/MSI-H, confirmed by NGS  Location: Right (14), Left (8)  Metastasis: 10 had liver met., 9 had peritoneal met.  **Lynch** Y:N = 10:12  **BRAF mut** Y:N = 4:18  **KRAS mut** Y:N = 9:13 | 19 patients treated with pembrolizumab monotherapy, 1 each received nivolumab, nivolumab/ipilimumab, and durvalumab/tremelimumab. | **Tumor mutation burden** high: TMB > 37~41 mutations/Mb  The number of synonymous and non-synonymous mutations across a 0.8- to 1.2-megabase region, with computational germline status filtering, detected with a hybrid capture based NGS assay.  TMB median (range): 47.5 (13-91) | Criteria: RECIST 1.1  Follow-up: 3 years  Potential biomarkers were analyzed with Cox regression and multivariant model. |
|  |  |  |  | **Liver Metastasis** |  |
| Publication year: 2020 | | | | | |
| Chen, E.X.  Canada  Multi-centered  Prospective Phase 2  NCT02870920  Group CO.26 study | Patients with confirmed adenocarcinoma of the colon or rectum, received all available standard systemic therapies. Not exposed to ICI before.  Age: median 65, range 39-87  Sex: Female: Male = 45:74 | pMMR/MSS, detected with baseline plasma for circulating cell-free DNA by GuardantOMNI NGS 2.15Mb, 500-gene panel.  Metastasis: 80 had liver met. | Treated with tremelimumab (anti-CTLA4) 75mg every 28days for the first 4 cycles plus durvalumab (anti-PD-L1) 1500mg every 28 days. | **Tumor mutation burden** high: TMB ≥ 28 varitations/Mb  All somatic synonymous and non-synonymous SNVs and indels excluding germline, clonal hematopoiesis of indeterminate potential, driver, and resistance variations, detected by GuardantOMNI NGS with circulating cell-free DNA in the baseline plasma. Results were statistically adjusted for sample-specific tumor shedding and molecular coverage.  TMB median (range): 15.3 (0.96-85.4) | Primary endpoints: OS  Criteria: RESICT 1.1  Radiographic assessments were performed every 8 weeks until progression.  Follow-up (median): 15.2 months |

| **Study** | **Characteristics of patients** | **Features of tumor** | **Treatment** | **Biomarker (Definition, test method, cutoffs)** | **Notes** |
| --- | --- | --- | --- | --- | --- |
| (continued) Publication year: 2020 | | | | | |
| Cohen, R  57, France  Multi-centered  Prospective  Phase 2  NCT03350126  NIPICOL | Patients with confirmed metastatic CRC locally assessed as dMMR/MSI. Not exposed to ICI before.  Age: median 56.5  Sex: Female: Male = 27:30  Previous treatment: one line (5), two (24), more (27), missing (1) | dMMR/MSI-H, confirmed with IHC and/or PCR testing. Tumors were designated as MSI if two or more markers were instable.  Location: Right (31), Left (25)  **Lynch** Y:N:U = 32: 16: 9  **BRAF mut** Y:N:U = 10: 28:19  **RAS mut** Y:N:U = 28: 10:19 | Received nivolumab 3mg/kg and ipilimumab 1mg/kg every 3 weeks for four cycles, and then nivolumab 3mg/kg every 2 weeks until disease progression. | **Neutrophil-to-lymphocyte ratio** high: NLR ≥ 3  A derived NLR was used  **Derived NLR = neutrophils/(leukocytes minus neutrophils)* | Primary endpoints: disease control rate at 12 weeks  Criteira: RECIST 1.1 and iRECIST. Radiographic assessments were performed every 6 weeks for 24 weeks and every 12 weeks thereafter.  Follow-up (median): 18.1 months |
| Fukuoka, S.  Japan,  Single-centered  Prospective  Phase 1b  NCT03406871 | Patients with histologically or cytologically confirmed advanced or metastatic solid tumors refractory to or intolerant of standard chemotherapy.  Age: median 55, range 31-77  Sex: Female: Male = 7:18 | dMMR:pMMR = 1:24, assessed by IHC of four MMR enzymes. Tumor that lacked either enzyme expression was considered MMR deficient. MSI was determined using Promega MSI kit.  Location: Right (5), Left (20)  Metastasis: 13 with liver met., 16 with lung met., and 4 with peritoneal met. | Regorafenib of 80-160 mg was administered once daily for 21 days on/7 days off with nivolumbab 3mg/kg every 2 weeks. | **PD-L1 expression** high: CPS ≥ 1  Combined positive score (CPS) is the number of PD-L1 positive cells (tumor cells, lymphocytes, and macrophages) as a proportion of the total number of tumor cells multiplied by 100, assessed centrally by a pathologist using the anti-PD-L1 28-8 antibody. | Primary endpoints: dose-limiting toxicity during the first 4 weeks  Criteria: RECIST 1.1  CT were performed every 8 weeks until progression.  Follow-up: 2 years |
|  |  |  |  | **Tumor mutation burden** high: TMB ≥ 10.9 mutations/Mb  Definition was not mentioned in the article. The value of was detected by the Oncomine Tumor Mutation Load Assay. The median TMB was used as the cutoff value.  ** Top and lower percentile was also used as cutoffs.* |  |
|  |  |  |  | **Liver metastasis** |  |
| Kawazoe, A.  Japan  Multi-centered  Prospective  Phase 1/2  NCT02851004 | Histologically confirmed metastatic CRC refractory to or intolerant of standard chemotherapy.  Age (range): 25-79  Sex: Female: Male = 28:22 Previous treatment: <=2 (18), more (32) | dMMR:pMMR = 10:40, assessed by IHC of four MMR proteins. Tumor that lacked either protein expression was considered dMMR. MSI status was determined using the Promega MSI kit  Location: Right (37), Left (13)  **BRAF^V600E^ mut** Y:N:U = 4:33:13  **RAS mut** Y:N:U = 30:19:1 | Napabucasin, a small molecule inhibitor of STAT3, plus pembrolizumab | **PD-L1 expression** high: CPS ≥ 1  Combined positive score (CPS) is the number of PD-L1 positive cells (tumor cells, lymphocytes, and macrophages) as a proportion of the total number of tumor cells multiplied by 100, assessed by a pathologist using the anti-PD-L1 IHC 22C3 pharmDx antibody. | Primary endpoint: immune-realted ORR  Criteria: RECIST 1.1or irRECIST  Radiological assessment was performed every 6 weeks until 24 weeks. |
| Li, J.S.  China, Shandong  Multi-centered  Retrospective | Patients with metastatic CRC refractory to standard therapy of at least two lines of chemotherapy +/-biologics. Not exposed to ICI before.  Age: median 50, range 33-73  Sex: Female: Male =7:16 | pMMR/MSS, determined by MMR protein IHC or PCR-based assay for microsatellite loci.  Location: Right colon (6), Left colon (7), Rectum (10)  **BRAF mut** Y:N:U = 1:12:10  **RAS mut** Y:N:U = 12:1:10 | Receiving an anti-PD-1 antibody (nivolumab, pembrolizumab, camrelizumab, sintilimab, toripalimab) combined with regorafenib (80-160 mg once per day for 3 weeks on/1 week off in 4-week cycles). | **Liver metastasis** | Criteria: RECIST 1.1 |
| Loupakis, F.  Italy  Mult-centered  Prospectively planned observational study | Metastatic CRC with tumor tissue specimen available.  Age: median 60, range 17-85  Sex: Female: Male = 35:45 | dMMR/MSI-H, centrally confirmed by means of PCR and sequencing.  Location: Right (56), Left (22)  Metastasis: 31 had multiple metastatic sites, 25 with liver met., 7 with lung met., and 30 with peritoneal met.  **BRAF mut** Y:N = 28:52  **RAS mut** Y:N = 27: 53 | Treated with ICIs (i.e., anti-PD-1 pembrolizumab or nivolumab and/or anti-CTLA-4 ipilimumab) | **Liver Metastasis** | Criteria: RECIST 1.1  Follow-up (median): 22.8 months |
|  |  |  |  | **Tumor-infiltrating lymphocytes** high: number of TILs ≥ 2.0  The number of TILs is defined as the mean value of five random observations and count and high-power fields (40×) of tumor-enriched areas composed of >60% of neoplastic cells, detected by IHC staining. Only tumor epithelium infiltrating lymphocytes were retained for scoring. |  |
| Publication year: 2021 | | | | | |
| Chida, K.  Japan  Multi-centered  Retrospective  2015 to 2020 | Histologically proven, unresectable, locally advanced, or metastatic GI tumor (refractory to or intolerant of one or more chemotherapy) | dMMR/MSI-H, verified by PCR or IHC testing. Tumors were classified as MSI-H if instability was noted in a minimum of two markers by Promega MSI analysis system. | Received an anti-PD-1 inhibitor allone or as combination therapy (pembrolizumab plus napabucasin) | **Tumor mutation burden** high: TMB ≥ 10 mutations/Mb  TMB was defined as the total number of nonsynonymous mutations, including indels, detected by WES, the Oncomine Cancer Research Panel (NGS), or FoundationOne Liquid. | Criteria: RECIST 1.1 |

| **Study** | **Characteristics of patients** | **Features of tumor** | **Treatment** | **Biomarker (Definition, test method, cutoffs)** | **Notes** |
| --- | --- | --- | --- | --- | --- |
| (continued) Publication year: 2021 | | | | | |
| Kim, D.W.  America  Single-centered  Prospective  Phase 1/2  NCT03332498 | Adults with confirmed MSS metastatic colorectal adenocarcinoma, refractory to or intolerant of standard chemotherapy  Age: median 59, range 24-73  Sex: Female: Male = 16:22  Race: White (30), Blacks (5) | pMMR/MSS, detection method not mentioned.  Location: Right (9), Left (29)  Metastasis: 27 with liver met., 25 with lung met., and 14 with peritoneum met.  **RAS mut** Y:N = 19:19 | Treated with oral ibrutinib once daily and a fixed dose of pembrolizumab 200mg intravenously every 3 weeks | **Neutrophil-to-lymphocyte ratio** high: NLR ≥ 5  The ratio was defined as the absolute neutrophil count divided by the absolute lymphocyte count, obtained from complete blood count with differential. | Primary endpoint: disease control rate at 4 months  Criteria: RECIST 1.1  Radiological assessment was performed every 9 weeks until disease progression or treatment discontinuation. |
| Kawazoe, A.  Japan  Multi-centered  Prospective  Phase 1b  NCT02851004  UMIN000032801 | Histologically or cytologically confirmed advanced or metastatic solid tumors refractory to or intolerant of standard chemotherapy.  Age: median 61, range 32-77  ** only for MSS CRC without prior anti-PD-1 treatment* | dMMR:pMMR = 4:25, assessed by IHC testing. Tumors that lacked either one of the 4 MMR protein were considered MMR deficient. MSI status was determined using the Promega MSI kit  **RAS mut (14), BRAF^V600E^ mut (6)** | TAS-116 (pimitespib), an oral HSP90 inhibitor, in combination with nivolumab (anti-PD-1 antibody) | **PD-L1 expression** high: CPS ≥ 1  Combined positive score (CPS) is the number of PD-L1 positive cells (tumor cells, lymphocytes, and macrophages) as a proportion of the total number of tumor cells multiplied by 100, assessed by a pathologist using the anti-PD-L1 IHC 22C3 pharmDx antibody. | Criteria: RECIST 1.1  Radiological assessment was performed every 6 weeks until 24 weeks (thereafter every 9 weeks until week 42…) |
|  |  |  |  | **Tumor mutation burden** TMB ≥ 9.6 mutations/Mb  TMB is measured from extracted DNA from archival tumor samples using the Oncomine Tumor Mutation Load Assay. The median TMB was used as the cutoff value. |  |
|  |  |  |  | **Liver Metastasis** |  |
| Parikh. A.R.  America  Single-centered  Prospective  Phase2  NCT03104439 | Metastatic CRC with confirmed MSS  Age: median 60, range 32-75  Sex: Female: Male = 7:18 | pMMR/MSS, confirmed by PCR and/or IHC | Combined dual blockade of PD-1 (nivolumab) and CTLA-4 (ipilimumab) with radiation therapy | **Tumor mutation burden** high: TMB ≥ 4.35 mutations/Mb  TMB was computed for (1) all mutations, (2) nonsynonymous SNV mutations, and (3) missense mutations, detected by WES.  TMB median (range): 4.35 (0.82-7.72) | Primary endpoint: disease control rate by intention to treat.  Criteria: RECIST 1.1  Follow up: 5 years |
| Sahin, I.H.  America  Multi-centered  Retrospective  2012 to 2019 | Patients with dMMR/MSI-H CRC, diagnosed at the range of stage II-IV.  Age: <50 (14), 50-65 (22), >65 (24)  Sex: Female: Male = 27:33  Previous treatment: no (15), one line (22), two (9), more (13), missing (1) | dMMR/MSI-H, confirmed with IHC or PCR conducted at each clinical center.  Location: Right (46), Left (14)  **BRAF mut** Y:N:U = 9:31:20  **RAS mut** Y:N:U = 11:14:35 | ICI single-agent (pembrolizumab or nivolumab) or ICI in combination (nivolumab and ipilimumab) were accepted. | **Liver metastasis** | Criteria: best objective response was evaluated respectively by investigators using RECIST 1.1, while progression was made by local physicians.  Follow-up (median): 28.3 months |
| Valero, C.  America  Retrospective  2016 to 2018 | Patients with response and survival outcomes after ICI treatment. | NA | Received anti-PD1 therapy or anti-PD-L1 therapy or their combo. | **Neutrophil-to-lymphocyte ratio** high: NLR ≥ 5  The ratio is calculated with the absolute counts of Neu and Lym, pretreatment, in peripheral blood.  ** Percentile was also used as cutoffs in the article.* | Follow-up: 18 months |
|  |  |  |  | **Tumor mutation burden** high: TMB ≥ 12 mutations/Mb  Definition was not mentioned in the article.  The value of TMB was derived from NGS.  TMB median (range): 7.9 (1.8-368.6) mutations/Mb |  |
| Wang. C.K.  American  Single-centered  Retrospective  2014 to 2020 | Patients with MSS metastatic CRC and refractory to standard chemotherapy  Age: median 55, IQR 49-64  Sex: Female: Male = 41:54 | pMMR/MSS, testing method not described in the article.  Location: Right (26), Left (69)  Metastasis: 54 with liver met., 66 with lung met., and 29 with peritoneum met.  **RAS mut** Y:N = 58:37**,**  **BRAF^V600E^ mut** Y:N = 4:91 | Received PD-1/PD-L1-targeting therapy, and other investigational agents were allowed to be used in combination. | **Liver metastasis** | Criteria: RECIST 1.1 |

| **Study** | **Characteristics of patients** | **Features of tumor** | **Treatment** | **Biomarker (Definition, test method, cutoffs)** | **Notes** |
| --- | --- | --- | --- | --- | --- |
| (continued) Publication year: 2021 | | | | | |
| Yang, K.L.  China,  Multi-centered  Retrospective  NCT04771715 | Patients with advanced or metastatic CRC, excluding patients with confirmed MSI-H/dMMR status.  Age: median 63, range 35-81  Sex: Female: Male = 34:50  Previous treatment: no (1), one (7), two (25), more (51) | pMMR/MSS, tested by four MMR protein IHC or PCR examining the five microsatellite loci.  Location: Right (20), Left (64)  Metastasis: 55 had liver met. 59 had multiple metastatic sites.  **BRAF^V600E^ mut** Y:N:U = 3:73:8  **RAS mut** Y:N:U = 45:31:8 | Treated with at least one dose of ICIs combined with regorafenib. The types of ICIs (anti PD-1), treatment doses and schedules were determined per investigator’s decision. | **Neutrophil-to-lymphocyte ratio** high: NLR ≥ 1.5  It is calculated from the baseline complete blood count results.  The cutoff value is defined using the maximally selected rank statistics method. | Criteria: RECIST 1.1  Follow-up (median): 5.5 months |
|  |  |  |  | **Liver Metastasis** |  |
| Zhou, C.  China,  Single-centered  Retrospective  2015 to 2020 | Patients from the First Affiliated Hospital of Sun Yat-sen University received ICIs therapy and underwent genomic profiling by WES. | dMMR/MSI-H, defined as an MSI score (the number of unstable microsatellite sites/ total valid sites) >3.5%. | Received ICIs (nivolumab, atezolizumab, pembrolizumab, ipilimumab, and toripalimab) as either a monotherapy or combination therapy. | **Tumor mutation burden** high: TMB ≥ 10 mutations/Mb  The total number of somatic non-synonymous SNVs and indels in the tumor exome, detected by WES. Somatic mutations in tumors were identified through comparison with peripheral blood from the same patient.  ** Top tertile was also used as cutoffs in the article.* | Follow-up: 12 months |
| Zhou. H  China, Fujian  Single-centered  Retrospective  2019 to2021 | Patients with metastatic CRC and received no prior systemic therapy (adjuvant therapy, radiotherapy, or surgery for mCRC were allowed).  Age: median 64, range 43-86  Sex: Female: Male = 12:13 | dMMR:pMMR = 4:21  Location: Right (7), Left (18) | Treated with camrelizumab (200mg on day1 every 3 weeks) combined with XELOX plus bevaxizumab (7.5 mg/kg on day 1 every 3 weeks) or regorafenib. | **Liver metastasis** | Criteria: RECIST 1.1, CT was performed every two or three cycles of the combination therapy. |
| Publication year: 2022 | | | | | |
| Bando, H.  Japan  Multi-centered  Prospective  Phase1/2  NCT02948348 | Treatment -naïve with rectal cancer located 12 cm from the anal verge, clinical stage T3-4 N0-2 M0, treated using capecitabine-based concurrent chemoradiotherapy | pMMR/MSS, detect method not mentioned in the article. | Included five cycles of nivolumab monotherapy (240mg every 2 weeks) and subsequent radical surgery. | **Tumor-infiltrating lymphocytes**  Tumor samples were detected by flow cytometry, the ratio of CD8+ T cells to regulatory T cells before preoperative chemoradiotherapy was analyzed. Based on ROC curve analysis, a ratio ≥ 2.5 was considered high | Primary endpoint: pathologic complete response rate  Criteria: AJCC 7^th^ edition. |
| Ciardiello, D.  Italy  Multi-centered  Prospective  Phase 2  NCT04561336  CAVE trial | RAS WT, histologically confirmed mCRC, should have obtained response during first line anti-EGFR chemotherapy, and should have progressed.  Age: median 63, range 54-69  Sex: Female: Male = 35:42  Previous treatment: two (52), three or more (25) | dMMR:pMMR = 3:71, with 3 patients whose MMR status were unknown.  Location: Right colon (5), Left colon (47), Rectum (25)  **RAS/BRAF mut:** Y:N:U = 19:48:10 | Received cetuximab at 400 mg/m^2^, as loading dose, and, subsequently, at 250 mg/m^2^ weekly, and avelumab was given intravenously at 10 mg/kg, once every 2 weeks. | **Neutrophil-to-lymphocyte ratio** high: NLR ≥ 3  It is defined as the absolute neutrophil count divided by the absolute lymphocyte count, obtained from complete blood count with differential. | Primary endpoint: OS  Criteria: RECIST 1.1,  Radiological assessment was performed at baseline, and every 8 weeks for 40 weeks and every 12 weeks thereafter. |
| Cheng, Y.K.  China  Multi-centered  Retrospective  2016 to 2019 | Patients suffered metastatic CRC with deficient MMR status and possessed flow cytometry results.  Age: median 41, range 20-77  Sex: Female: Male = 19:22 | dMMR/MSI-H, test method was not mentioned.  Location: Colon (30), Rectum (11)  **BRAF mut** Y:N:U = 2:20:19  **KRAS mut** Y:N:U = 16:6:19 | Treated with anti-PD1 inhibitor (nivolumab, pembrolizumab, triprizumab, toripalimab, or camrelizumab). | **Neutrophil-to-lymphocyte ratio** high: NLR ≥ 4  It is defined by the absolute counts of Neu and Lym, pre-treatment.  The cutoff value is defined using the ROC curve analysis | Criteria: RESICT 1.1  Potential biomarkers were analyzed with Cox regression and multivariant model. |
|  |  |  |  | **Tumor-infiltrating lymphocytes**  Detected by flow cytometry, the ratio of CD4+/CD8+ T cells (cutoff 1.64), the frequency of CD4+T cells (cutoff 39.5%) and CD8+ T cells was analyzed.  ** also analyzed as continuous variable in the article* |  |

| **Study** | **Characteristics of patients** | **Features of tumor** | **Treatment** | **Biomarker (Definition, test method, cutoffs)** | **Notes** |
| --- | --- | --- | --- | --- | --- |
| (continued) Publication year: 2022 | | | | | |
| Garralada, E.  America  Single-centered  Prospective  Phase 1  NCT02720068  The MK-4280-001 study | Participants had to have MSS locally advanced or mCRC that progressed on all available standard-of-care therapies without prior anti-PD-1/PD-L1 therapy  Age: median 58, range 32-81  Sex: Female: Male = 17:54 | pMMR/MSS. MSI-H (2 of 5 microsatellite markers changed) or dMMR (1 of 4 proteins lost) status was determined locally by PCR or IHC before or during screening. | Received 800mg favezelimab (Anti-LAG3) monotherapy, followed by 800mg favezelimab plus 200mg pembrolizumab either in sequential administered or as the co-formulation. | **PD-L1 expression** high: CPS ≥ 1  Measured with combined proportion score (CPS), which is not defined in the article. a PD-L1 22C3 IHC staining were performed but not required to be completed in the study design. | Primary endpoints: safety and tolerability  Criteria: RECIST 1.1  Radiological assessment (CT/MRI) was performed at 9 weeks after first dose and every 9 weeks thereafter. |
| Hyung, J.  Korea  Single-centered  Retrospective  2015 to 2020 | Patients diagnosed with dMMR/MSI-H colorectal adenocarcinoma and finished the study treatment were included. | dMMR/MSI-H, tested by MMR protein IHC or targeted NGS. MSI-H was determined by a TMB more than 40 and an indels mutation to whole mutation percentage more than 9% | Treated with pembrolizumab 200mg every 3 weeks, avelumab 10 mg/kg every 2 weeks, or durvalumab 1,500mg every 4 weeks. | **PD-L1 expression** high: CPS ≥ 1  Measured with combined proportion score (CPS), which is defined as the ratio of all PD-L1-positive cells to viable tumor cells. PD-L1 22C3 IHC staining were performed and the results with CPS was interpreted by one pathologist. | Criteria for respond is that patients who received ICI treatment for > 4 months without progression after 2 consecutive disease evaluations within 8 to 9 weeks. |
| Kim, R.D.  America  Single-centered  Prospective  Phase 1/1b  NCT03712943 | Histologically confirmed pMMR metastatic colorectal adenocarcinoma, refractory or intolerant to standard chemotherapy +/- biologics.  Age: median 56, range 31-79  Sex: Female: Male = 24:28  Race: White (42), Asian (4), African American (3), Hispanic (3)  Previous treatment: two lines (30), more (22) | pMMR/MSS, test method not mentioned.  Location: Right (30), Left (22)  Metastasis: 38 with liver met., 33 with lung met., and 11 with peritoneal met..  **RAS mut** Y:N = 37:15 | Regorafenib 80mg daily plus nivolumab 240 mg. | **PD-L1 expression** high: positive  Positivity was defined as the samples with ≥ 1% tumor cells with PD-L1 membranous staining at any intensity, detected by IHC. | Primary endpoints: dose-limiting toxicity and maximum tolerated dose.  Criteria: RECIST 1.1,  CT was performed at baseline and every 8 weeks until disease progression or treatment discontinuation. |
|  |  |  |  | **Liver metastasis** |  |
|  |  |  |  | **Tumor-infiltrating lymphocytes**  Detected by IHC, the frequency of CD8+T cells and regulatory T cells was analyzed. Samples with any lymphocytes with membranous CD4 and CD8 staining and intracellular FOXP3 staining were positive. |  |
| Li, R.R.  China, Hunan  Single-centered  Retrospective  2019 to 2021 | CRC patients with at least second-line treatment  Age: median 56, range 20-79  Sex: Female: Male = 47:56 | pMMR/MSI, tested by IHC, PCR or NGS panel validation.  Location: Right colon (24), Left colon (27), Rectum (52)  Metastasis: 66 patients had multiple metastasis, 59 with liver met., 45 with lung met., and 14 with peritoneal met. | Treated with at least one dose of anti-PD-1 antibodies (nivolumab, pembrolizumab, camrelizumab, sintilimab, toripalimab) plus regorafenib (80mg once daily for 21 days on/7 days off 28 days as a cycle) | **Liver metastasis** | Primary endpoints: OS  Criteira: RECIST 1.1,  CT was performed every 2 or 3 treatment cycles until progression or being lost to follow-up.  Follow-up: 2 years |
| Mettu, N.B.  America  Multi-centered  Prospective  Phase 2  NCT02873195  The BACCI study | Adult patients with metastatic CRC who experienced disease progression while receiving chemotherapy +/- bevacizumab. Not exposed to ICI before.  Age: median 59, range 53-66  Sex: Female: Male = 35: 47  Race: White (66), Asian (6), Black (7), Others (3) | dMMR:pMMR = 9:69, with 4 missing data on MMR status. MMR status were determined by local testing at individual sites.  Location: Colon (57), Rectum (25)  **RAS mut** Y:N = 50:32 | Capecitabine (850 or 1000 mg/m^2^) twice daily on days 1 to 14 and bevacizumab on day 1 plus atezolizumab (1200mg) on day 1 of each 21-day cycle. | **Liver metastasis** | Primary endpoint: PFS  Criteria: RECIST 1.1, assessment were performed every 12 weeks or as clinically indicated.  Follow-up (median): 20.9 months |
| Xu, Y.J.  China  Single-centered  Retrospective  2018 to 2020 | Histologically or cytologically proven with MSS mCRC treated with more than two lines of standard chemotherapy regimens.  Age (range): 27-73  Sex: Female: Male = 14:16 | pMMR/MSS, detected by MMR protein IHC or PCR for microsatellite loci. The MMR/MSI status was evaluated by the 2B3D method.  Location: Colon (16), Rectum (14)  **BRAF mut** Y:N:U = 0:19:11  **KRAS mut** Y:N:U = 7:12:11 | During a 4-wk treatment cycle, regorafenib was performed for 3 continuous weeks. PD-1 inhibitor was intravenously injected starting on the first day of the oral intake of regorafenib. | **Liver metastasis** | Criteria: RECIST 1.1 |
| **Study** | **Characteristics of patients** | **Features of tumor** | **Treatment** | **Biomarker (Definition, test method, cutoffs)** | **Notes** |
| (continued) Publication year: 2022 | | | | | |
| Wang, Y  China  Single-centered  Retrospective  2011 to 2017 | Patients with CRC at Beijing Cancer Hosptial.  Age: median 44, range 14-75  Sex: Female: Male = 7:14 | dMMR:pMMR = 16:2, with 3 unknown, detected by IHC or PCR. Instability at two or more of the markers with the same method was considered dMMR/MSI. | Treated with PD1/PD-L1 inhibitor with or without a CTLA-4 inhibitor. | **Tumor mutation burden** high: TMB ≥ 37 mutations/Mb  Defined as the number of synonymous and non- synonymous mutations. Test method not mentioned in the article.  TMB median (range): 38.1 (2.3-220.3) mutations/Mb | Criteria: RECIST 1.1 |
| Publication year: 2023 | | | | | |
| Fakih, M.  America  Single-centered  Prospective  phase 1  NCT04362839 | Patients with MSS metastatic CRC that had progressed on or after standard treatment. Not exposed to regorafenib or anti-PD therapy before.  Age: median 55, range 36-75  Sex: Female: Male = 17:12  Race: White (21) Asian (4), Black (3)  Previous therapy: one (1), two (14), more (14) | pMMR/MSS, detection method not mentioned in the article.  Location: Right (9), Left (20)  **BRAF mut (Y)**:3 ; **RAS mut (Y)**: 21 | 80 mg once daily for 21 days every 28-day cycle, along with ipilimumab, 1 mg/kg, intravenously every 6 weeks and nivolumab, 240 mg, intravenously every 2 weeks | **Liver metastasis** | Primary endpoint: RP2D selection  Criteria: both RECIST 1.1 and iRECIST 1.1.  Radiological assessment was performed within 14 days before the first dose and every 8 weeks. |
| Fakih, M.  America  Multi-centered  Prospective  Phase 2  NCT04126733 | Adults with previously treated advanced MSS/pMMR mCRC. They had known extended RAS and BRAF status, progression or intolerance to no more than three line of systemic chemotherapy.  Age: median 57, range 50-66  Sex: Female: Male = 29:41  Race: White (50), Asian (7), Black (10) | pMMR/MSS, detection method not mentioned in the article  Location: Right (25), Left (45)  Metastasis: 47 with liver met., 51 with lung met., 6 with peritoneal met.  **BRAF mut** Y:N:U = 3:65:2  **RAS mut** Y:N:U = 43:25:2 | Regorafenib 80mg/day was administered orally for 3 weeks on/1 week off with intravenous nivolumab 480 mg every 4 weeks | **Liver metastasis** | Primary endpoint: ORR  Criteria: RECIST 1.1.  Radiological assessments were carried out every 8 weeks for the first year, and every 12 weeks thereafter. |
| Jung, J  Korea  Sinle-centered  Retrospective  2019 to 2021 | The genomic profiles of patients with solid cancer who underwent the TSO500 assay. | NA | PD-L1 therapy. | **Tumor mutation burden** high ≥ 10 mutations/Mb  TMB is calculated by the total number of somatic alterations (both non-synonymous and synonymous SNVs and indels in the coding region with a variant frequency ≥5%), identified by WES. The effective size for TMB calculation was the total coding region with coverage >50×. | / |
| Manca, P.  Italy  Multi-centered  Retrospective  2016 to 2022 | Patients with dMMR and/or MSI-H metastatic CRC.  Age: <70 (69), >=70 (41)  Sex: Female: Male = 57:53  Previous therapy: none (46), others (64) | dMMR/MSI-H, assessed locally by means of IHC and/or PCR, respectively, as per international guidelines  Location: Right (88), Left (22)  Metastasis: 32 with liver met., 16 with lung met., 46 with peritoneal met., 56 had multiple metastases  **BRAF mut (Y)**:48; **RAS mut (Y)**: 19  **Lynch**: Y:N:U = 25:69:16 | 30 patients received an anti-CTLA-4-based combination, whereas the remaining received anti-PD-(L)1 monotherapy | **Tumor mutation burden** high ≥ 23 mutations/Mb  It is the total number of mutations per coding area of the tumor genome, detected by comprehensive genomic profiling (CPG) with primary or metastatic tumor sample. | Criteria: RECIST 1.1 |
| Moretto, R.  Italy  Multi-centered  Prospective  Phase 2  NCT03721653  AtezoTRIBE | Patients with initially unresectable metastatic CRC | pMMR/MSS, as the analysis were separately performed in the pMMR subgroup, detection methods not mentioned in the article | Received FOLFOXIRI/bev plus the anti-PD-L1 atezolizumab (840 mg intravenously). All treatment were administered up to 8 14-day cylces, followed by maintenance with 5-FU and leuvocorin plus bev with atezolizumab. | **PD-L1 expression** high: positive  Positivity was defined as the samples with ≥ 1% tumor cells with PD-L1, detected by IHC and quantified by digital pathology. with surgically resected specimens or biopsies from either primary tumor or metastatic sites | Criteria: RECIST 1.1 |
|  |  |  |  | **Tumor-infiltrating lymphocytes** high: average TILs ≥ 2.0  The density of TILs was defined as the mean value of five random observations and counts at high-power fields (40X) of tumor-enriched areas made of >60% of neoplastic cells on HE-stained sections, detected by optical microscope and assessed by two pathologists. |  |
| **Study** | **Characteristics of patients** | **Features of tumor** | **Treatment** | **Biomarker (Definition, test method, cutoffs)** | **Notes** |
| (continued) Publication year: 2023 | | | | | |
| Saberzadeh-Ardestani, B.  America  Multi-centered  Retrospective  2015 to 2022 | Patients (Elderly) with dMMR or MSI-H metastatic colorectal adenocarcinoma were identified from the electronic health record  Age: median 81, range 76-86  Sex: Female: Male = 29:12 | dMMR/MSI-H, detected by IHC, PCR or NGS with primary tumors. With a tumor mutation burden of 51 (IQR: 37-73) mut/Mb  Location: Right (30), Left (10), synchronous (1)  **Metastasis:** 14 with liver involvement, 12 with lung involvement  **KRAS mut** Y:N:U = 2:35:4**,**  **BRAF^V600E^ mut** Y:N = 30:8:3  **Lynch** Y:N = 8:32:1 | Pembrolizumab, 200mg was administered every 3 weeks | **Liver metastasis** | Primary endpoint: PFS  Criteria: RECIST 1.1 Follow-up (median):23 months  The biomarkers were confirmed with stepwise Cox proportional hazards regression modeling. |
| mCRC: metastatic colorectal cancer, d/p MMR: deficient/proficient mismatch repair protein, MSI: microsatellite instability, MSS: microsatellite stable, WES: whole exome sequencing, NGS: next generation sequencing, IHC: immunohistochemical staining, SNV: single nucleotide variants, ORR: objective response rate, OS: overall survival, PFS: progression-free survival, RECIST: Response Evaluation Criteria in Solid Tumours | | | | | |

**Supplementary Table 3**

**Quality Assessment with Newcastle-Ottawa Scale**

|  | Selection | | | | Comparability | | Outcome | | | scale |
| --- | --- | --- | --- | --- | --- | --- | --- | --- | --- | --- |
| DTLe2015 | * | * | * | * | * | * | * | * | * | 9 |
| BMONeil2017 |  |  | * | * |  |  | * | * | * | 5 |
| MJOverman2017 | * | * | * | * | * |  | * | * | * | 8 |
| ABSchrock2019 | * | * | * |  | * | * | * | * | * | 8 |
| EXChen2020 | * | * | * | * | * | * | * | * | * | 9 |
| RCohen2020 | * | * | * | * | * | * | * | * | * | 9 |
| SFukuoka2020 | * | * | * | * |  |  | * | * | * | 7 |
| AKawazoe2020 | * | * | * | * | * |  | * | * | * | 8 |
| JSLi2020 | * | * | * |  | * |  | * | * | * | 7 |
| FLoupakis2020 | * | * | * | * | * | * | * | * | * | 9 |
| KChida2021 | * | * | * |  | * |  | * | * | * | 8 |
| AKawazoe2021 | * | * | * | * |  |  | * | * | * | 7 |
| DWKim2021 | * | * | * | * | * |  | * | * | * | 8 |
| ARParikh2021 |  | * | * | * | * |  | * | * |  | 6 |
| IHSahin2021 | * | * | * |  | * | * | * | * | * | 8 |
| CValero2021 |  | * | * |  |  | * |  | * | * | 5 |
| CKWang2021 | * | * | * |  | * | * | * | * | * | 8 |
| KLYang2021 | * | * | * |  | * |  | * | * | * | 7 |
| CZhou2021 |  | * | * |  | * | * |  | * |  | 5 |
| HZhou2021 |  | * | * |  |  |  | * | * | * | 5 |
| HBando2022 |  | * | * | * | * | * | * | * | * | 8 |
| YKCheng2021 | * | * | * |  | * | * | * | * | * | 8 |
| DCiardiello2022 |  | * | * | * |  | * | * | * | * | 7 |
| JHyung2022 | * | * | * |  | * |  | * | * | * | 7 |
| RDKim2022 | * | * | * | * | * | * | * | * | * | 9 |
| RRLi2022 | * | * | * |  | * |  | * | * | * | 7 |
| NBMettu2022 |  | * | * | * |  | * | * | * | * | 7 |
| YJXu2022 | * | * | * |  | * | * | * | * | * | 8 |
| YNWang2022 | * | * | * |  |  | * | * | * | * | 7 |
| EGarralda2022 | * | * | * | * | * |  | * | * | * | 8 |
| PManca2023 | * | * | * |  | * |  | * | * | * | 8 |
| MFakih-12023 | * | * | * | * | * | * | * | * | * | 9 |
| MFakih-22023 | * | * | * | * | * | * | * | * | * | 9 |
| JJung2023 | * | * | * |  |  |  |  | * | * | 5 |
| RMoretto2023 | * | * | * | * | * |  | * | * | * | 8 |
| ABSaberzdeh2023 | * | * | * |  | * | * | * | * | * | 8 |

**Acronym Table for this review**

| **Acronym** | **Full name** |
| --- | --- |
| BTKi | Bruton’s tyrosine kinase inhibitor |
| CI | confidence interval |
| CPS | combined positive score |
| CR | complete response |
| CTLA-4 | cytotoxic T-lymphocyte-associated protein 4 |
| CRC | colorectal cancer |
| dMMR | deficient mismatch-repair gene |
| HR | hazard ratio |
| HSP90i | heat shock protein 90 inhibitor |
| ICI | immune checkpoint inhibitor |
| LAG-3 | lymphocyte-activation gene 3 |
| LM | liver metastasis |
| MSI-H | microsatellite instability-high |
| MSS | microsatellite stable |
| NLR | neutrophil-to-lymphocyte ratio |
| NOS | Newcastle-Ottawa scale |
| OR | odds ratio |
| ORR | objective response rate |
| OS | overall survival |
| PD1 | programmed cell death protein 1 |
| PD-L1 | programmed death ligand 1 |
| PFS | progression-free survival |
| pMMR | proficient mismatch-repair gene |
| PR | partial response |
| STATi | signal transducer and activator of transcription inhibitor |
| TIL | tumor-infiltrating lymphocyte |
| TMB | tumor mutation burden |
| VEGFRi | vascular endothelial growth factor receptor inhibitor |

**Supplementary Figure 1 Sensitivity Analysis**


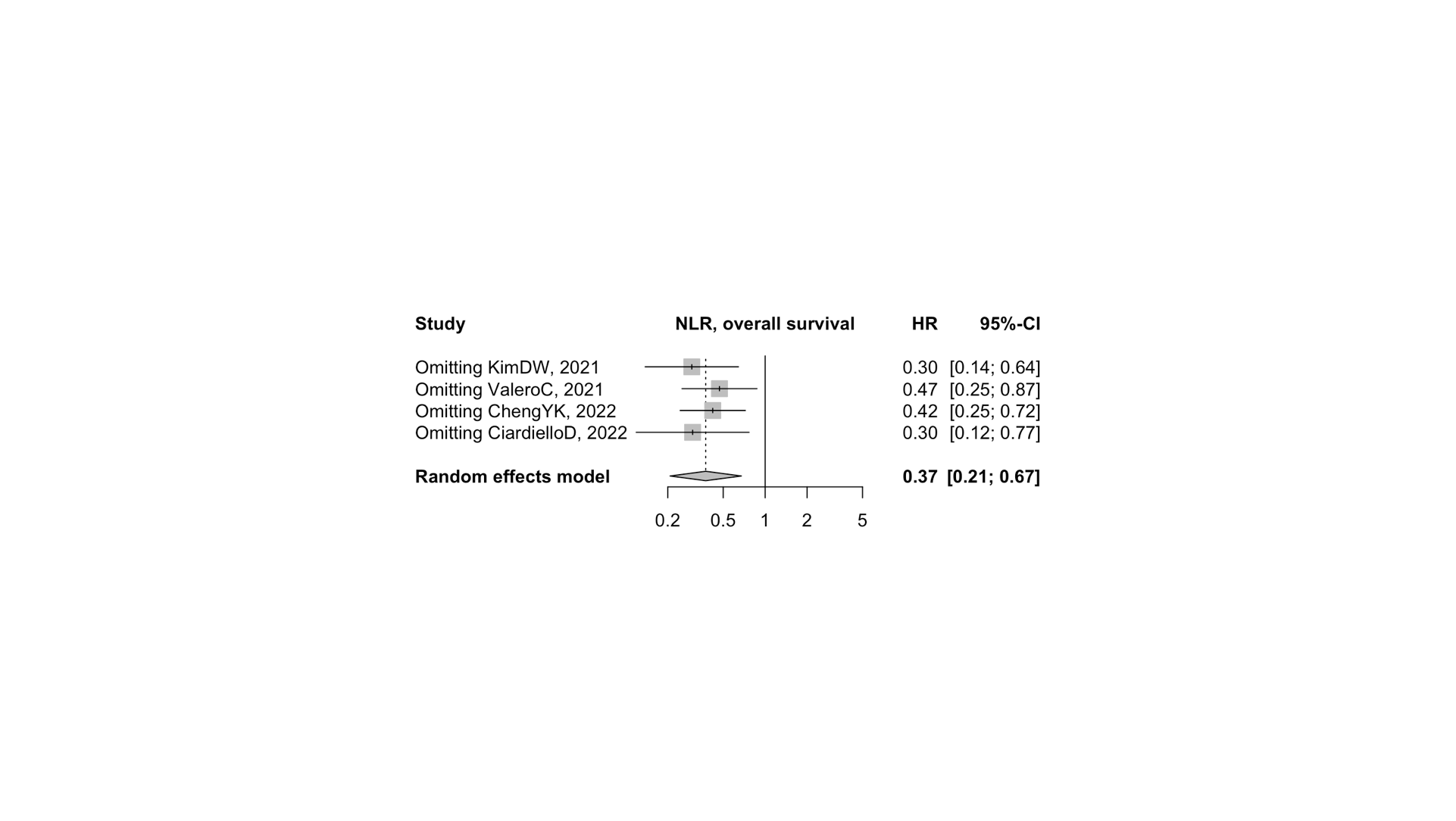
A


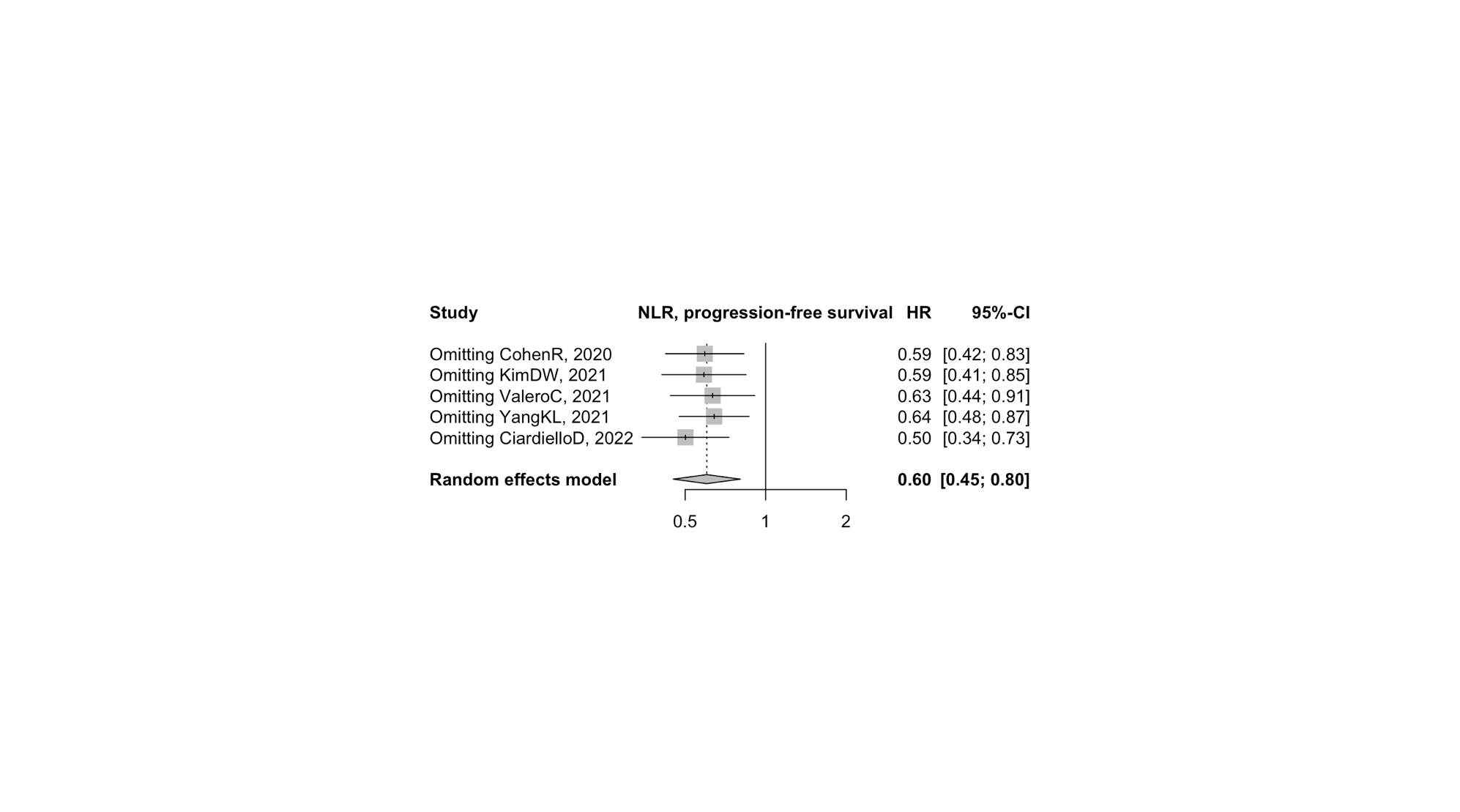


B


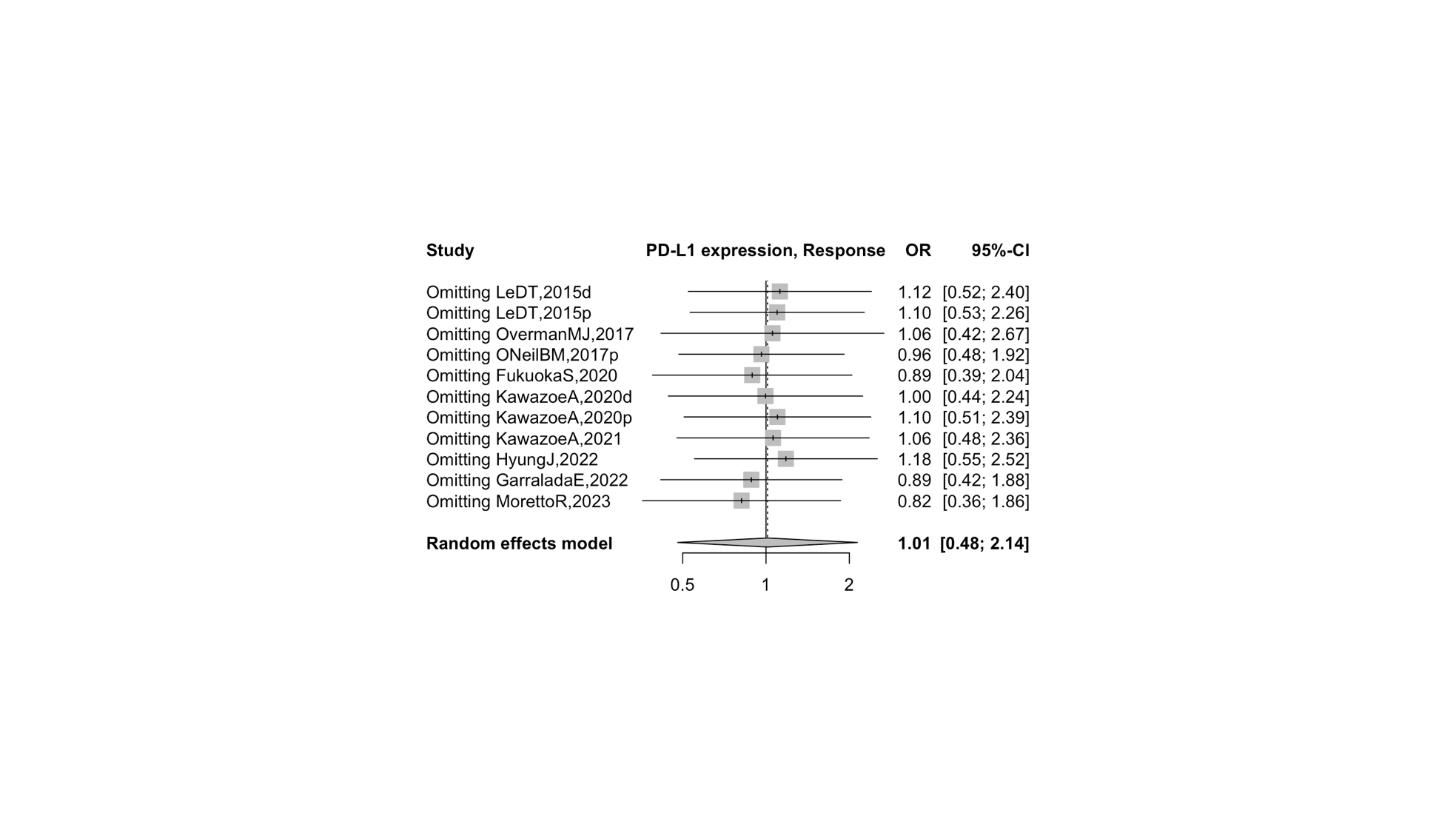


C


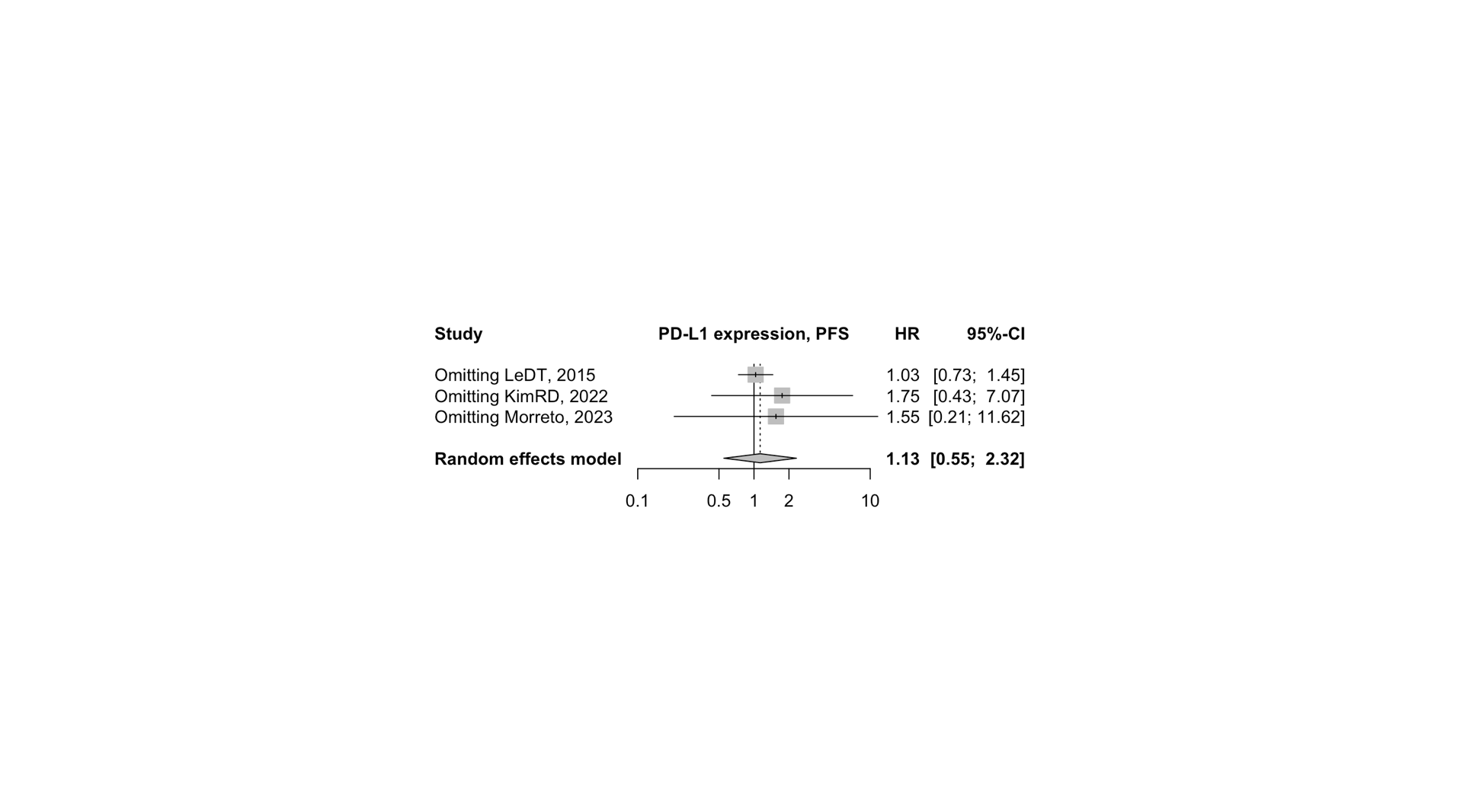


D


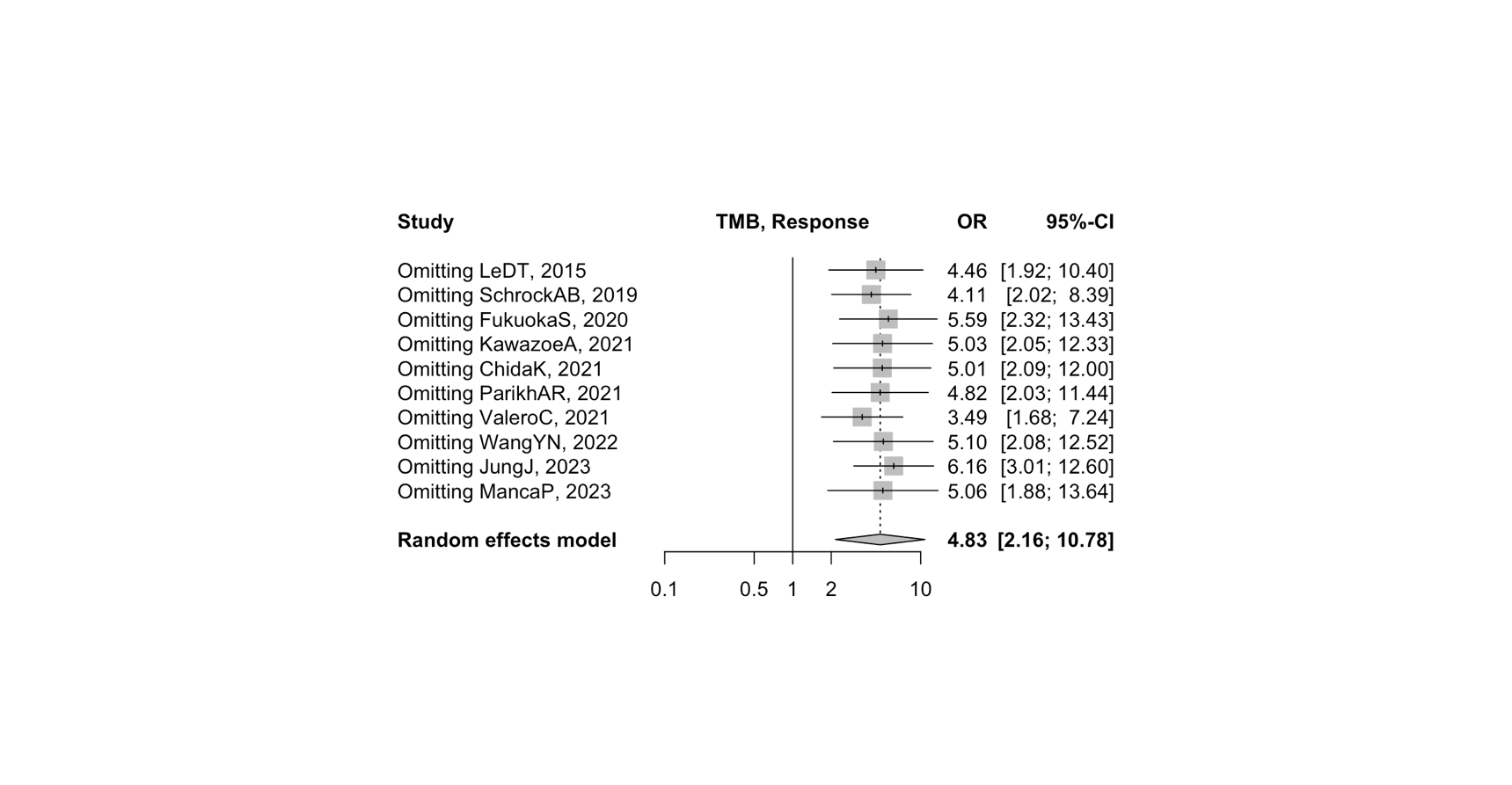


E

**Supplementary Figure 1.** Sensitivity analysis for neutrophil-to-lymphocyte ratio (NLR) on overall survival (A), for NLR on progression-free survival (B). Sensitivity analysis for PD-L1 expression on response (C), for PD-L1 expression on progression-free survival (D). Sensitivity analysis for tumor mutation burden (TMB) on response (E).

**Supplementary Figure 2 Results of liver metastasis**


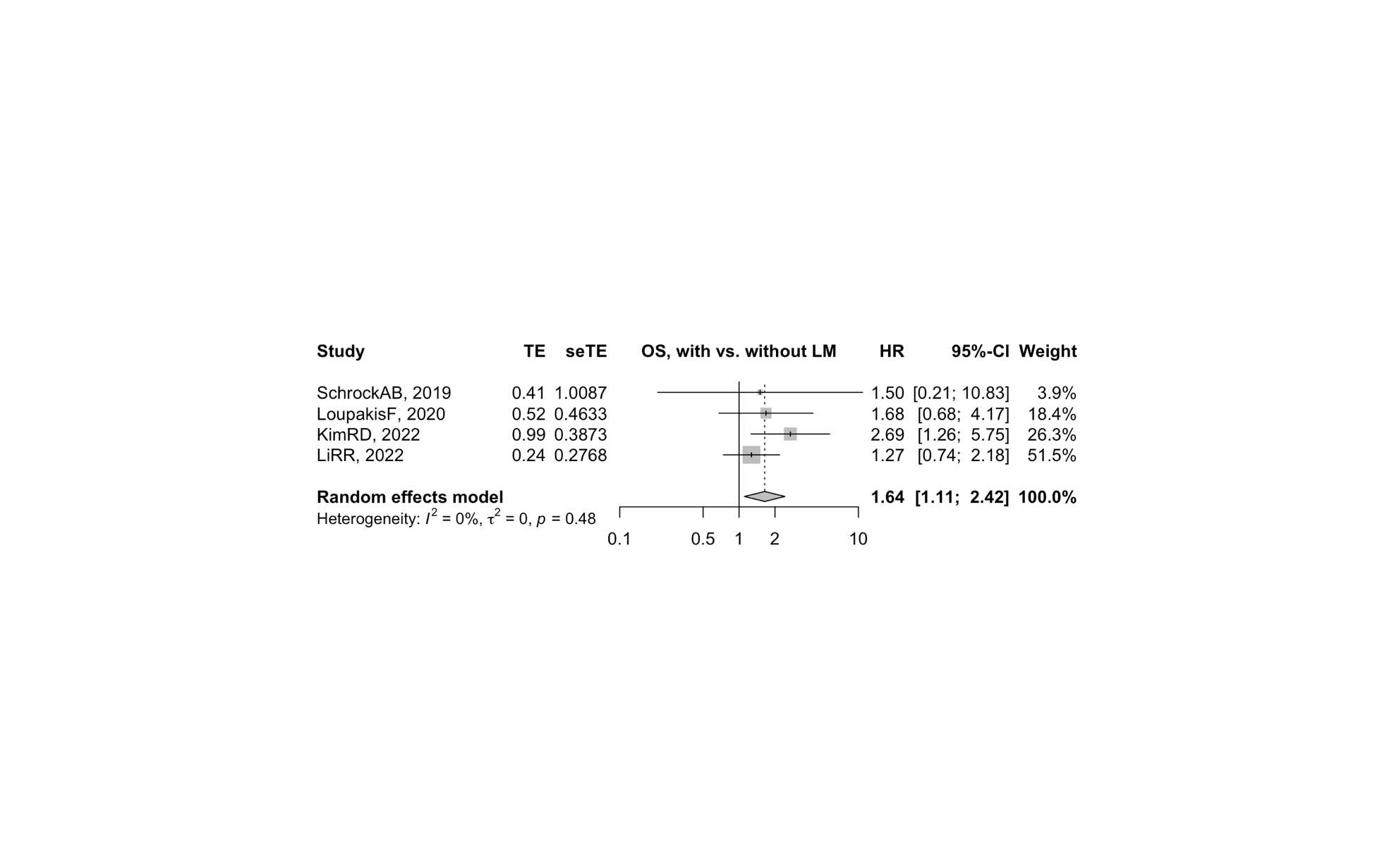
A

B D


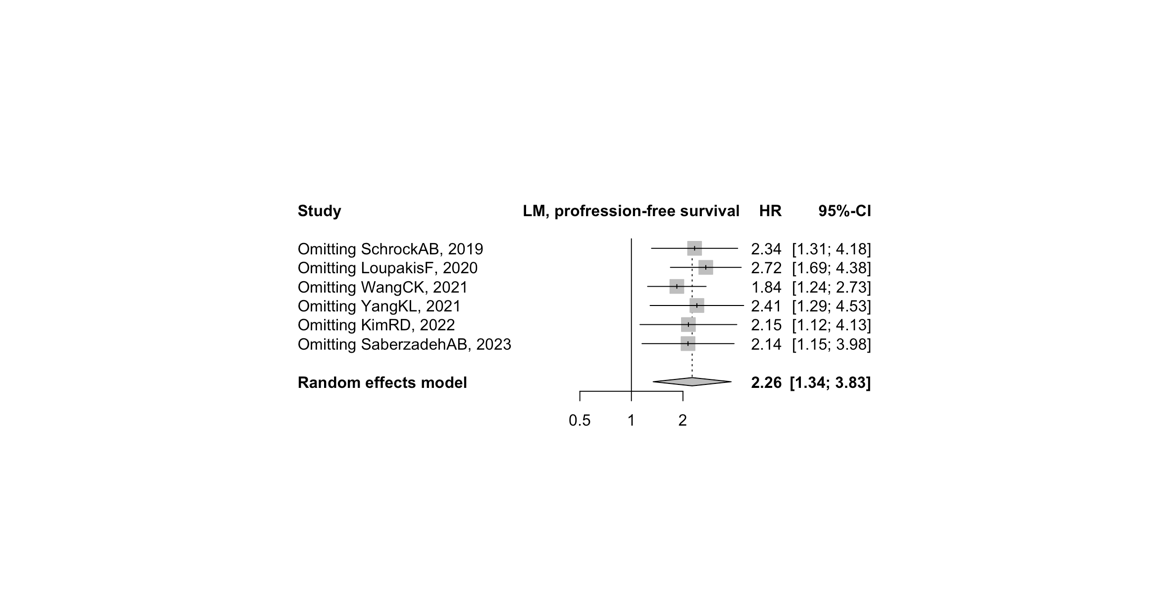

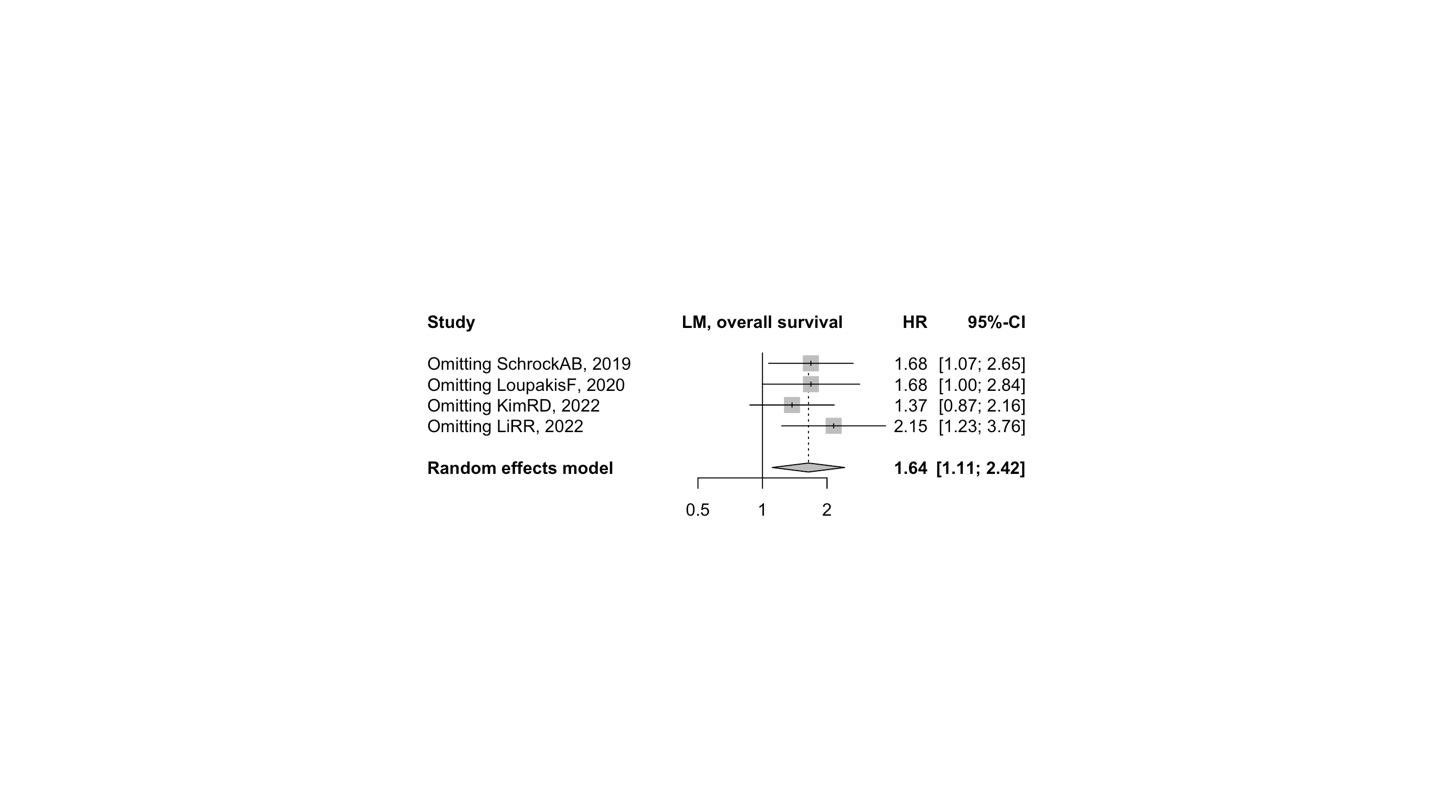


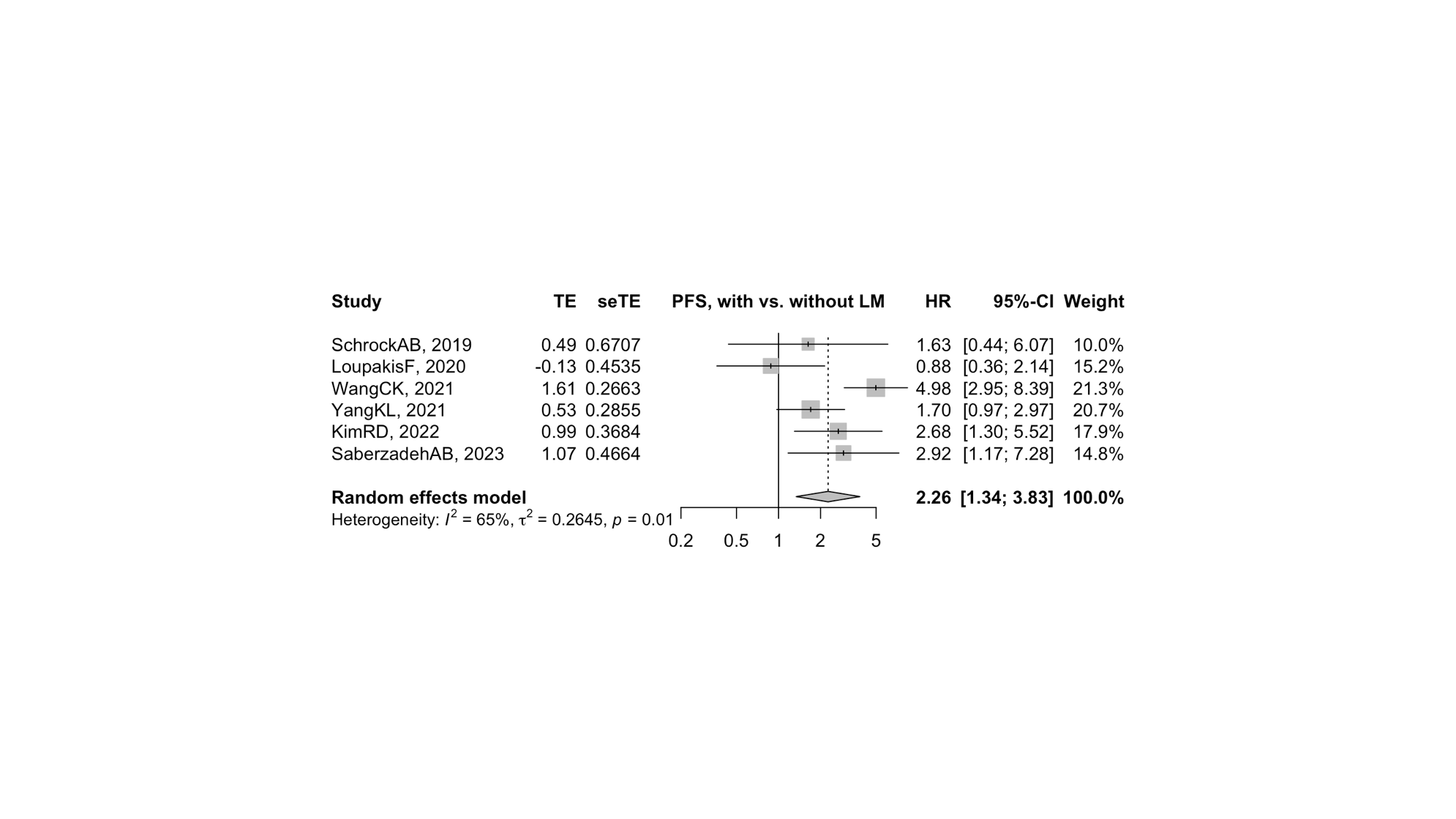
C

E


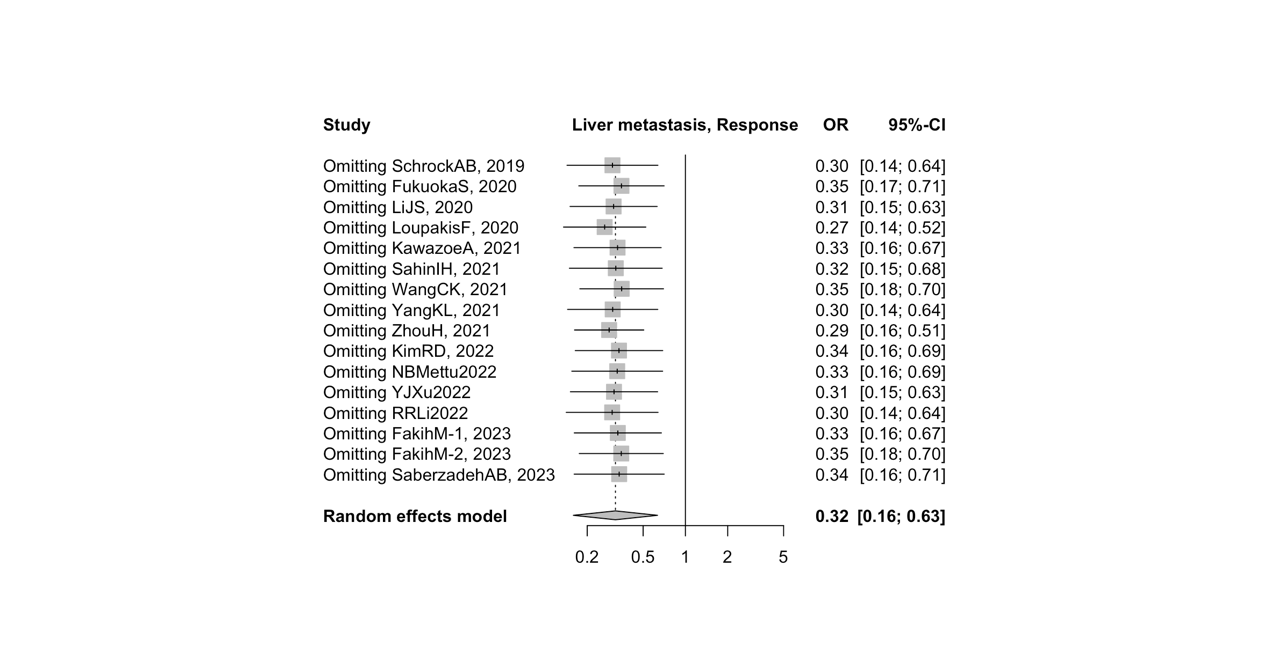


**Supplementary Figure 2**. Forest plot for liver metastasis on overall survival (A), and its sensitivity analysis (B). Forest plot for liver metastasis on progression-free survival (C), and its sensitivity analysis (D). The sensitivity analysis for liver metastasis on response (E).
